# SP1‐Mediated Glycolytic Reprogramming Promotes Tumorigenesis and Progression in Pancreatic Cancer

**DOI:** 10.1002/advs.202510071

**Published:** 2025-08-20

**Authors:** Hexing Hang, Mengyu Yu, Linxi Zhu, Neng Tang, Xiao Fu, Zhenghua Cai, Minghao Yan, Yi Chen, Lei Yang, Jianzhuang Wu, Jiatong Tang, Yu Xie, Qi Li, Xu Fu, Liang Mao, Jun Chen, Fanqing Meng, Bo Kong, Xiaodong Han, Chao Yan, Yudong Qiu, Hao Cheng

**Affiliations:** ^1^ Department of Pancreatic and Metabolic Surgery Nanjing Drum Tower Hospital Affiliated Hospital of Medical School Nanjing University Nanjing Jiangsu 210008 China; ^2^ Department of Pathology Nanjing Drum Tower Hospital Affiliated Hospital of Medical School Nanjing University Nanjing Jiangsu 210008 China; ^3^ Jiangsu Key Laboratory of Molecular Medicine Medical School Nanjing University Nanjing Jiangsu 210093 China; ^4^ The Affiliated Jiangning Hospital of Nanjing Medical University Nanjing Jiangsu 211100 China; ^5^ Immunology and Reproduction Biology Laboratory & State Key Laboratory of Analytical Chemistry for Life Science Medical School Nanjing University Nanjing Jiangsu 210093 China; ^6^ Clinical and Translational Research Center Affiliated Hospital of Nantong University & Department of Oncology Medical School of Nantong University Nantong Jiangsu 226007 China; ^7^ State Key Laboratory of Pharmaceutical Biotechnology School of life Sciences Nanjing University Nanjing Jiangsu 210023 China; ^8^ Department of General Visceral and Transplantation Surgery University of Heidelberg 69117 Heidelberg Germany; ^9^ Chemistry and Biomedicine Innovation Center Institute of Artificial Intelligence Biomedicine Nanjing University Nanjing Jiangsu 210034 China; ^10^ Institute of Pancreatology Nanjing University Nanjing Jiangsu 210008 China

**Keywords:** PanIN, PDAC, SP1, metabolic remodeling, pancreatic cancer

## Abstract

Pancreatic ductal adenocarcinoma (PDAC) is a highly lethal malignancy, commonly progressing from pancreatic intraepithelial neoplasia (PanIN). However, the molecular alterations in PanIN lesions and their contribution to PDAC progression remain poorly defined. Here, using laser capture microdissection‐based proteomics of patient tissues, early metabolic remodeling and upregulation of the transcriptional factor specificity protein 1 (SP1) in PanIN lesions are identified, which persisted into the PDAC stage. That SP1 overexpression promoted PDAC proliferation is demonstrated in patient‐derived organoid xenograft models (PDOXs), while deletion of Sp1 inhibited tumorigenesis and progression in a transgenic mouse model of PDAC (*Kras^LSL‐G12D/+^; Trp53^LSL‐R172H/+^; Sp1^LOXP/LOXP^; Pdx1‐Cre*). ChIP‐seq and isotope tracing revealed that SP1 enhances aerobic glycolysis by transcriptionally activating 6‐phosphofructo‐2‐kinase/fructose‐2,6‐bisphosphatase (PFKFB4), a key regulator of glycolysis. Combination therapy targeting SP1 and PFKFB4 demonstrated significant efficacy in PDAC models in vivo. The findings suggest that SP1 is a critical regulator of PDAC initiation and progression through its control of metabolic remodeling. Targeting SP1 and PFKFB4 represents a promising therapeutic strategy for PDAC treatment.

## Graphical abstract

Laser capture microdissection‐based proteomics of pancreatic intraepithelial neoplasia (PanIN) identifies specificity protein 1 (SP1) as a key driver of tumorigenesis and progression in pancreatic cancer through glycolytic remodeling. SP1 enhances aerobic glycolysis by transcriptionally activating 6‐phosphofructo‐2‐kinase/fructose‐2,6‐bisphosphatase (PFKFB4). Combination therapy targeting SP1 and PFKFB4 demonstrates significant efficacy in pancreatic cancer models in vivo, without observable toxicity.

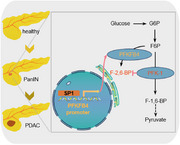

## 1 Introduction

Pancreatic cancer is the fourth leading cause of cancer‐associated mortality in both men and women,^[^ ^1^ ^]^ with a devastating 5‐year survival rate of less than 12%, and is projected to become the second leading cause by 2030.^[^ ^2^ ^]^ Pancreatic ductal adenocarcinomas (PDACs) comprise ≈90% of pancreatic cancers.^[^ ^3^ ^]^ The lack of effective screening tools for detecting asymptomatic premalignant or early‐stage carcinomas is a major contributor to the poor prognosis associated with PDAC. Most patients exhibit metastatic dissemination upon initial diagnosis. A multicenter study on early pancreatic cancer revealed a favorable prognosis for early‐stage PDAC patients following surgical resection;^[^ ^4^ ^]^ underscoring the importance of understanding how PDACs evolve from precursor lesions to invasive tumors.

Pancreatic intraepithelial neoplasia (PanIN) is the predominant precursor lesion associated with pancreatic cancer,^[^ ^5^ ^]^ which can be classified into grades 1–3 based on cytological atypia. ≈66% of somatic mutations detected in PDAC are also present in adjacent PanIN tissues,^[^ ^6^ ^]^ suggesting an evolutionary route from PanIN to PDAC. The mean latency between the detection of high‐grade PanIN and the diagnosis of PDAC is estimated to be ≈4 years, providing a crucial window for early screening and intervention.^[^ ^7^ ^]^ This critical window is currently untapped due to the absence of reliable early biomarkers beyond late‐stage indicators like carbohydrate antigen 19‐9 (CA19‐9).^[^ ^8^ ^]^ Identifying the molecular events driving PanIN initiation and progression to PDAC could facilitate the discovery of biomarkers for early diagnosis and the development of novel therapeutics for both early and late‐stage PDAC.

While transcriptomic studies have laid the groundwork for comparing gene expression between PanIN and PDAC samples,^[^ ^9^, ^10^ ^]^ proteomic analyses of human PanIN remain limited, primarily due to the scarcity of PanIN samples and the technical challenges associated with sample processing. While normal adult pancreas harbours hundreds of PanINs carrying oncogenic *KRAS* mutations, their minute size imposes significant research challenges.^[^ ^11^ ^]^ Metabolic remodeling – a hallmark of advanced PDAC driven by mutant *KRAS* ^[^ ^12^ ^]^ – may theoretically initiate during the PanIN stage, but evidence has been limited by stromal dilution in conventional bulk tissue analyses. Bulk proteomic analyses of PDAC tissues fail to resolve PanIN‐specific changes due to stromal contamination, while laser capture microdissection (LCM) – a spatially resolved technology‐ overcomes this barrier by dissecting <0.1 mm^2^ lesions precisely, enabling proteomic profiling of pure PanIN lesions from formalin‐fixed paraffin‐embedding (FFPE) samples.^[^ ^13^, ^14^ ^]^ By excluding stromal interference, LCM preserves authentic proteomic profiles, thereby providing an indispensable tool for identifying drivers of early pancreatic carcinogenesis.

In this study, we performed high‐resolution proteomic analysis on five human PanIN samples, isolated via LCM, alongside adjacent normal pancreatic tissues, to identify proteins and pathways involved in the initiation and progression of PDAC. Our results revealed metabolic remodeling within PanIN tissues, a phenomenon previously reported in PDAC but not in PanIN. Further analysis of transcription factors regulating these metabolic changes identified the overexpression of specificity protein 1 (SP1) in PanINs. SP1 is a known regulator of proliferation, angiogenesis, and metastasis in various tumors, including osteosarcoma, gastric carcinoma, and glioblastoma.^[^ ^15^, ^16^, ^17^ ^]^ Metabolic remodeling is a defining feature of advanced PDAC, driven primarily by mutant *KRAS* and characterized by enhanced glycolysis and macropinocytosis,^[^ ^12^, ^18^ ^]^ whether SP1 coordinates metabolic remodeling in PanIN lesions remains unexplored – a gap this study addresses by integrating LCM‐based proteomics on human PanIN lesions and functional validation in transgenic mice. In our study, SP1 knockout or knockdown inhibited tumorigenesis in genetically engineered mouse models and suppressed the growth of PDAC patient‐derived organoids (PDO) in vivo. Mechanistically, we demonstrated that SP1 transcriptionally activated the key glycolytic enzyme 6‐phosphofructo‐2‐kinase/fructose‐2,6‐bisphosphatase (PFKFB4), thereby regulating aerobic glycolysis, which maintains tumor growth and survival.^[^ ^12^ ^]^ These findings demonstrated that metabolic remodeling occurs early, before the onset of advanced PDAC, and plays a critical role in tumorigenesis and tumor progression.

## 2 Results

### 2.1 LCM‐Based Proteomics Reveal Metabolic Remodeling in Pancreatic Intraepithelial Neoplasia (PanIN)

**Figure 1:**
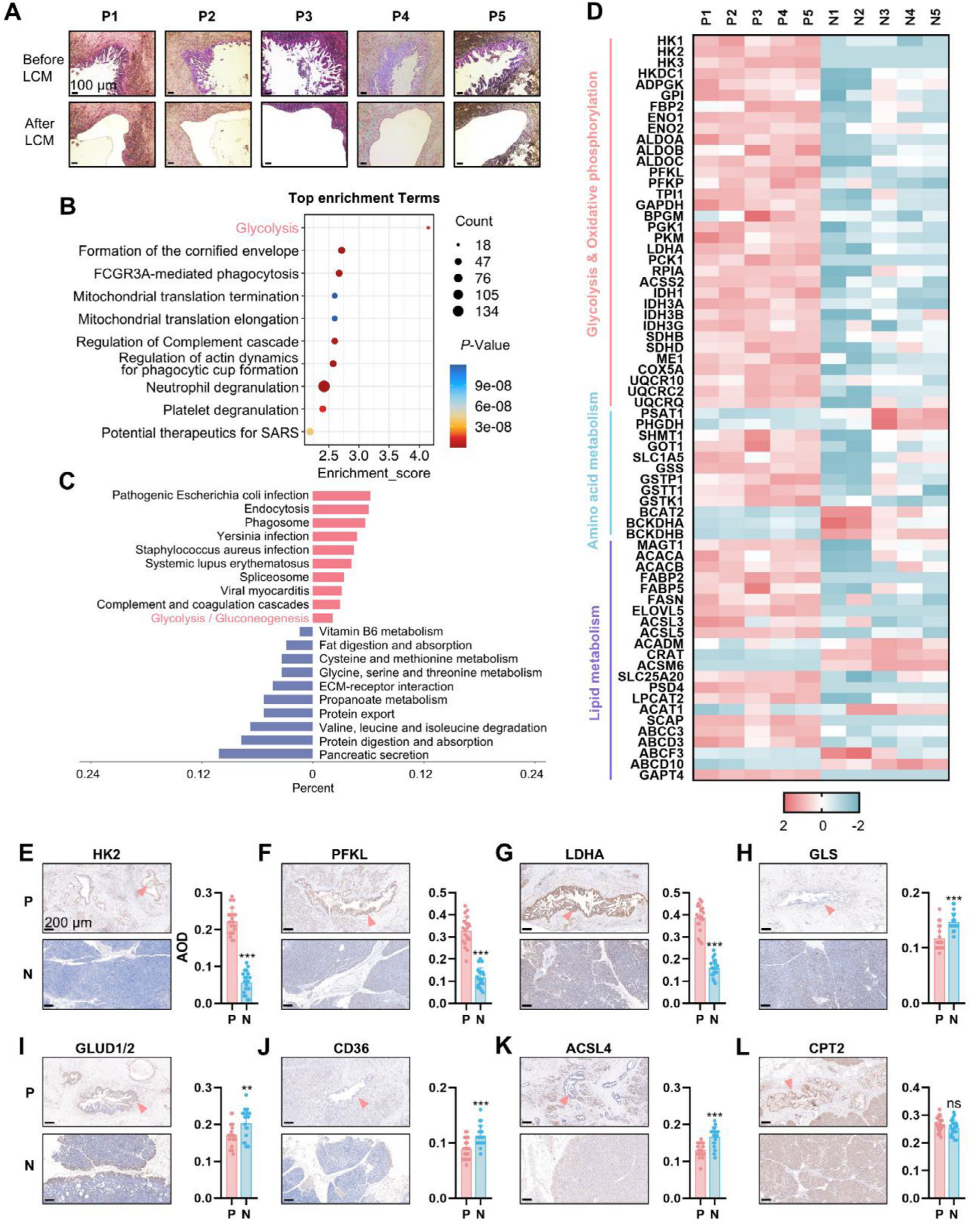
Metabolic remodeling in PanIN tissues. A) Laser capture microdissection of PanIN lesions from five PDAC patients. HE staining before and after LCM is shown. Scale bars, 100 µm. B, C) Reactome (B) and KEGG (C) pathway enrichment analysis of differentially expressed proteins between PanIN tissues and adjacent normal tissues. D) Heatmap of differentially expressed metabolic enzymes. (E‐L) Immunohistochemistry (IHC) staining and quantification of HK2, PFKL, LDHA, GLS, GLUD1/2, CD36, ACSL4, CPT2 in PanIN tissues (*n* = 20) and adjacent normal pancreatic tissues (*n* = 20). *P*‐value by Paired Student's t‐test. Scale bars, 200 µm. Data are shown as mean ± SD. **p < 0.01 and ***p < 0.001.

It is well established that PDAC tumor cells undergo metabolic remodeling to adapt to the hypoxic and nutrient‐deficient tumor microenvironment.^[^ ^12^, ^19^, ^20^, ^21^ ^]^ However, whether such changes occur during the PanIN stage remains unclear. To systematically explore the metabolic changes at the PanIN stage, we performed proteomic studies on five PanIN (P) tissues and paired adjacent normal pancreatic tissues (N) from hematoxylin‐eosin (HE)‐stained paraffin‐embedded tissue sections. PanIN and normal tissues were respectively isolated from the tissue slices using the Laser Capture Microdissection (LCM) technique (**Figure** **1**A), followed by analysis on a timsTOF Pro2 mass spectrometry platform (Figure S1A,B, Supporting Information).

A total of 2600 differentially expressed proteins were identified between PanIN and normal tissues, with 2203 proteins upregulated and 397 proteins downregulated in PanINs (Figure S1C,D, Supporting Information). To further understand the biological functions of these differentially expressed proteins, Reactome, Kyoto Encyclopedia of Genes and Genomes (KEGG), and Gene Ontology (GO) enrichment analyses were performed (Figure 1B,C; Figure S1E, Supporting Information). All three analyses revealed significant upregulation of glycolysis‐related pathways in PanINs, a change also observed in PDACs.^[^ ^22^, ^23^ ^]^ In contrast, pathways associated with vitamin, lipid, and amino acid metabolism were down‐regulated in PanINs, a pattern not seen in PDACs. Among the top differentially expressed proteins, most glycolytic enzymes were upregulated in PanINs, whereas enzymes related to lipid and amino acid metabolism showed mixed results (Figure 1D).

To verify the observed metabolic alterations in PanIN, we collected PanIN tissues and adjacent normal pancreatic tissues from 20 PDAC patients and performed immunohistochemical staining against a panel of classical metabolic enzymes. Compared with normal pancreatic tissues from the same patients, PanIN tissues exhibited significant upregulation of HK2, PFKL, and LDHA, alongside downregulation of GLS, GLUD1/2, CD36, and ACSL4, suggesting enhanced glycolysis in PanIN (Figure 1E–L).

We also noticed the enrichment of pathways related to endocytosis, mitochondrial translation, and fructose‐1,6‐bisphosphate metabolism in PanINs (Figure 1B,C; Figure S1E, Supporting Information). Similar processes have been identified as key features of PDACs. For example, mutant *KRAS* in PDAC cells facilitates the uptake of extracellular nutrients such as glucose, serum lipids, and proteins via a specific endocytosis process known as macropinocytosis;^[^ ^24^ ^]^ PDACs also exhibit high levels of autophagy, where autophagosomes engulf and degrade protein aggregates in lysosomes to furnish intermediary metabolites for biosynthesis and energy production in mitochondria.^[^ ^25^ ^]^

Together, these findings demonstrate significant metabolic remodeling in PanIN tissues from PDAC patients and that enhanced glycolysis occurs as early as the PanIN stage of PDAC tumorigenesis.

### 2.2 SP1 Upregulation in PanINs and PDACs Correlates with Poor Patient Prognosis

**Figure 2:**
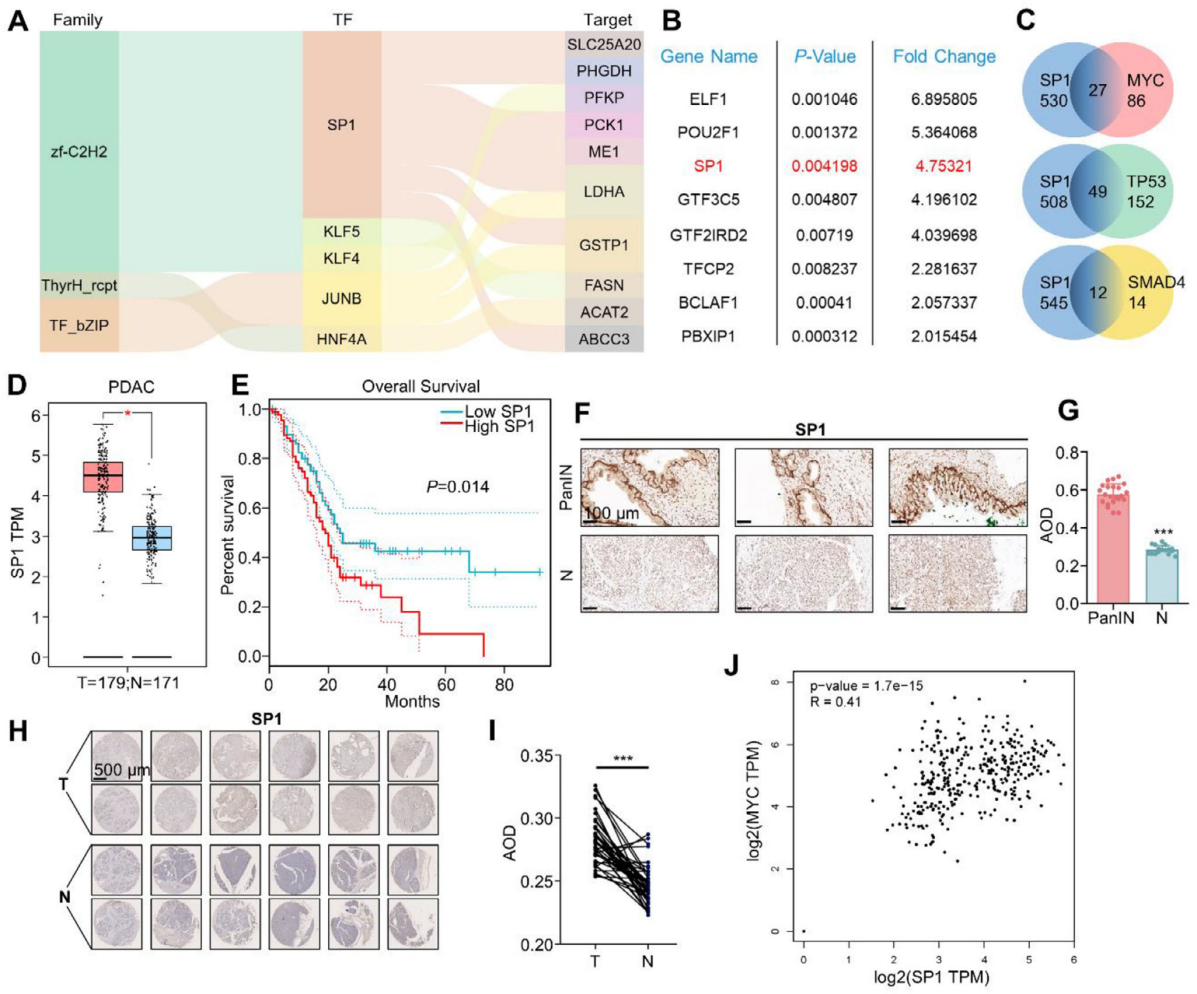
SP1 is overexpressed in PanIN and PDAC tissues. A) The Sankey diagram of differently expressed transcriptional factors and metabolic enzymes between PanIN tissues and adjacent normal tissues. B) The list of the most significantly up‐regulated transcription factors in PanIN tissues. C) Venn diagram showing the number of shared targets between SP1 and MYC, TP53, and SMAD4 using TRRUST. D) Box plot comparing the expression of SP1 in tumor and normal tissues of PDAC patients from The Cancer Genome Atlas (TCGA) and Genotype‐Tissue Expression (GTEx) datasets on GEPIA. *P*‐value by one‐way ANOVA test. E) Kaplan‐Meier curve showing overall survival (OS) of SP1‐high and SP1‐low PDAC patients from the TCGA dataset. *P*‐value by log‐rank test. F, G) Immunohistochemistry of SP1 in PanIN and normal pancreatic tissues (*n* = 22). Average optical density (AOD) analyzed by ImageJ. *P*‐value by Paired Student's t‐test. Scale bars, 100 µm. H, I) Immunohistochemistry of SP1 in a tissue array of tumor and normal pancreatic tissues from PDAC patients (*n *= 40). AOD was analyzed by ImageJ. *P*‐value by Paired Student's t‐test. Scale bars, 500 µm. J) Correlation analysis between the expression levels of SP1 and MYC from TCGA and GTEx datasets. *P*‐value by Spearman test. Data are shown as mean ± SD. *p < 0.05 and ***p < 0.001.

To investigate the transcriptomic regulatory network driving the metabolic remodeling of PanINs, we performed an integrative analysis of the expression levels of transcription factors and metabolic enzymes in our proteomics dataset. The Sankey diagram illustrating the differentially expressed transcriptional factors and their associated metabolic enzymes is shown in **Figure** **2**A. Five transcriptional factors – SP1, KLF4, KLF5, JUNB, and HNF4A – were identified as the most significant regulators responsible for the observed metabolic changes. Among these, SP1 was identified as one of the eight most significantly upregulated transcription factors in PanIN tissue (Figure 2B).

To evaluate the involvement of SP1 in PDAC tumorigenesis, we analyzed its interactions with three key driver genes of PDAC – MYC, TP53, and SMAD4 – using the Transcriptional Regulatory Relationships Unraveled by Sentence‐based Text Mining (TRRUST) tool,^[^ ^26^ ^]^ a manually curated database of human and mouse transcriptional regulatory networks. TRRUST analysis indicated that SP1 shared 27 target genes with MYC, 49 target genes with TP53, and 12 target genes with SMAD4 (Figure 2C). Moreover, data from the GEPIA database^[^ ^27^ ^]^ showed that SP1 expression was also higher in PDAC tissues compared to normal tissues (Figure 2D), suggesting its potential involvement in tumor progression.

Kaplan‐Meier survival analysis of PDAC patients revealed that, among the eight transcription factors significantly upregulated in PanIN tissue, only SP1 expression was inversely correlated with patient survival (Figure 2E; Figure S2B–H, Supporting Information). To confirm the upregulation of SP1 during PDAC initiation and progression, we performed immunohistochemical staining for SP1 in 22 PanIN tissues (Figure 2F,G) and in a tissue microarray composed of 40 PDAC tissues and paired adjacent normal tissues (Figure 2H,I). SP1 expression was significantly higher in both PanIN and PDAC tissues compared to normal pancreatic tissues (Figure 2F‐I). Furthermore, SP1 expression was positively correlated with the expression of MYC, KRAS, TP53, and SMAD4 in PDAC patients (Figure 2J; Figure S2I–K, Supporting Information). Notably, overexpression of SP1 was also observed in other malignant tumors, such as glioblastoma and stomach adenocarcinoma (Figure S2A, Supporting Information). Taken together, these transcriptomic data indicate that SP1 is upregulated at both the PanIN and PDAC stages and is associated with poor patient prognosis.

### 2.3 SP1 Promotes PDAC Tumor Growth

**Figure 3:**
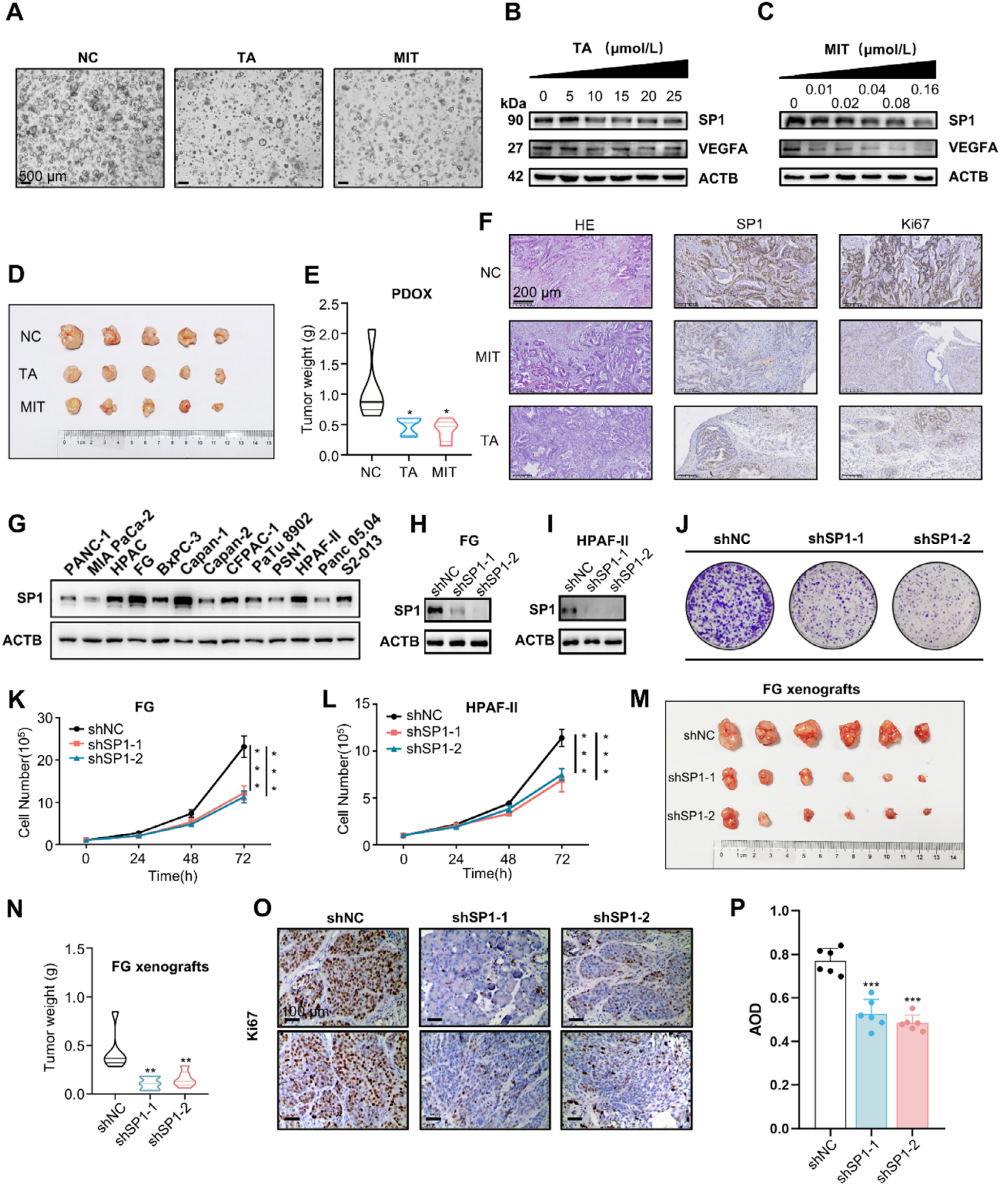
SP1 promotes the proliferation of pancreatic cancer. A) Representative images of PDAC patient‐derived organoids (PDO) – untreated or treated with SP1 inhibitors. Scale bars, 500 µm. (B, C) Effect of TA or MIT treatment on the protein levels of SP1 and VEGFA in the PDAC PDO model in vitro. (D, E) Effect of MIT or TA treatment on the growth of PDAC PDO in vivo. PDOs were orthotopically implanted into the pancreas of NCG mice, which were then treated with TA (gavage, 80 mg kg^−1^, every 3 days) or MIT (intraperitoneal injection, 1.5 mg kg^−1^, every 3 days) for 28 days. Tumor images were shown in D, and tumor weight in E (*n* = 5 mice per group). *P*‐value by one‐way ANOVA test. F) Representative images of HE and IHC staining of SP1 and Ki67 in PDOX tumor tissues. Scale bars, 200 µm. G) Protein level of SP1 in PANC‐1, MIA PaCa‐2, HPAC, FG, BxPC‐3, Capan‐1, Capan‐2, CFPAC‐1, PaTu 8902, PSN1, HPAF‐II, Panc 05.04 and S2‐013 cell lines. (H‐P) Knockdown of SP1 in FG and HPAF‐II cells inhibited tumor growth in vitro and in vivo. H, I) Immunoblotting of SP1 protein in FG and HPAF‐II cells showing the successful knockdown of SP1 by shRNA. J) Representative images of colony formation of control and SP1 knockdown FG cells. K, L) Growth curve of FG and HPAF‐II cells with or without SP1 knockdown. *p*‐value by two‐way ANOVA test. M, N) Effect of SP1 knockdown on the growth of FG cells orthotopically xenografted onto the pancreas of nude mice (*n* = 6 mice per group). Tumor weight was measured 5 weeks after transplantation. *P*‐value by one‐way ANOVA test. O, P) Representative images and AOD of Ki67 staining of FG CDX tumor tissues. Data are shown as mean ± SD. *p < 0.05, **p < 0.01 and ***p < 0.001.

To investigate the functional role of SP1 in PDAC, we tested the effect of SP1 inhibitors in a patient‐derived organoid (PDO) model of PDAC. PDOs were treated in vitro with mithramycin A (MIT) or tolfenamic acid (TA) (**Figure** **3**A), both of which have been reported to inhibit SP1 expression and its downstream target VEGFA.^[^ ^28^, ^29^ ^]^ Immunoblotting analysis showed that both MIT and TA effectively decreased SP1 and VEGFA protein levels in a dose‐dependent manner (Figure 3B,C; Figure S3A,B, Supporting Information). To further validate these findings in vivo, we orthotopically transplanted PDOs into the pancreas of NOD/ShiLtJGpt‐Prkdc^em26Cd52^Il2rg^em26Cd22^/Gpt (NCG) mice to establish a PDO xenograft (PDOX) model. Treatment of tumor‐bearing NCG mice with TA (gavage) or MIT (intraperitoneal injection) significantly inhibited the growth of PDOX tumors in vivo (Figure 3D,E). Immunohistochemical staining of the PDOX tumors after treatment revealed a reduction in SP1 and Ki67 levels (Figure 3F).

Additionally, we assessed SP1 expression across a panel of 13 PDAC cell lines (Figure 3G) and evaluated the effect of SP1 knockdown on the proliferation of these cell lines both in vitro and in vivo. Lentivirus‐mediated shRNA knockdown of SP1 expression significantly suppressed the proliferation and colony formation of FG and HPAF‐II cells in vitro (Figure 3H–L; Figure S3C,D, Supporting Information). SP1 knockdown also inhibited the proliferation of two other PDAC cell lines (Figure S3G,H, Supporting Information), suggesting a widespread effect of SP1 in PDAC cells. To assess the in vivo effects, parental and SP1‐knockdown FG cells were orthotopically transplanted into the pancreas of BALB/cNj‐Foxn1^nu^/Gpt (nude) mice to generate cell‐derived xenograft (CDX) models. Mice were sacrificed after 5weeks, and tumor tissues were analyzed. SP1 knockdown markedly inhibited the growth of FG and HPAF‐II cells in vivo (Figure 3M–P; Figure S3E,F, Supporting Information). Taken together, these results demonstrate that SP1 promotes the proliferation of PDAC both in vitro and in vivo.

### 2.4 SP1 Knockout Inhibits PDAC Tumorigenesis in *Kras^LSL‐G12D/+^; Trp53^LSL‐R172H/+^; Pdx1‐Cre* (KPC) Mice

**Figure 4:**
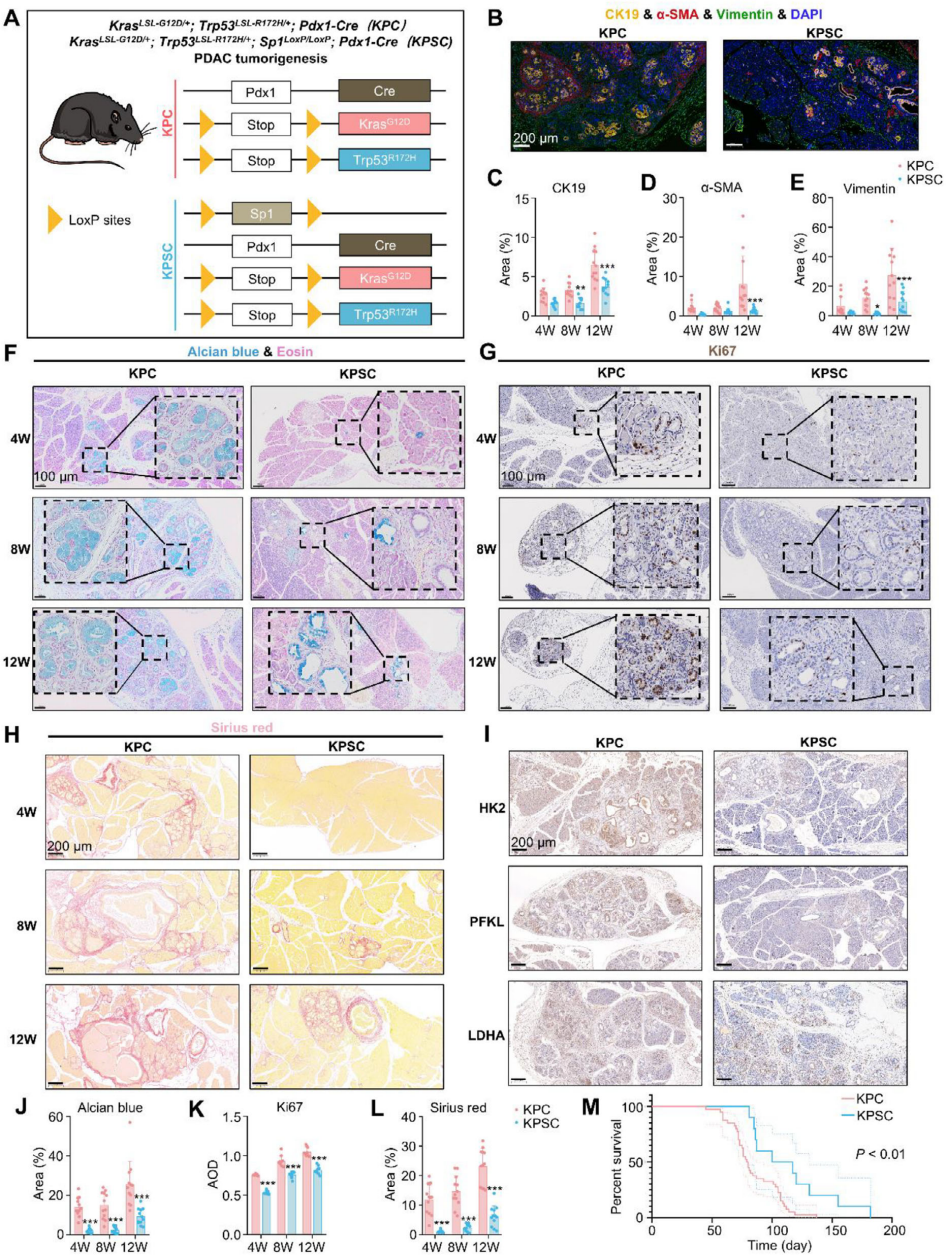
SP1 promotes tumorigenesis and progression of PDAC in *Kras^LSL‐G12D/+^; Trp53^LSL‐R172H/+^; Pdx1‐Cre* (KPC) mice. A) Schematic of establishing *Kras^LSL‐G12D/+^; Trp53^LSL‐R172H/+^; Sp1^LoxP/LoxP^; Pdx1‐Cre* (KPSC) and KPC mice. B) Representative images of immunofluorescence staining against CK19, α‐SMA, and Vimentin in the pancreas from KPC and KPSC mice (*n* = 3 mice per group) at 12 weeks. Scale bars, 200 µm. (C‐E) Quantification of the percentage of affected area of CK19, α‐SMA, and Vimentin in the pancreas slices from KPC and KPSC mice (*n* = 3 mice per group) at 4, 8, and 12 weeks. *P*‐value by two‐way ANOVA test. (F) Representative images of alcian blue staining of KPC and KPSC pancreas (*n* = 3 mice per group) at 4, 8, and 12 weeks. Scale bars, 100 µm. (G) Representative images of Ki67 staining of KPC and KPSC pancreas (*n* = 3 mice per group) at 4, 8, and 12 weeks. Scale bars, 100 µm. (H) Representative images of Sirius red staining of KPC and KPSC pancreas (*n* = 3 mice per group) at 4, 8, and 12 weeks. Scale bars, 200 µm. (I) Representative images of HK2, PFKL, and LDHA staining of KPC and KPSC pancreas (*n* = 3 mice per group) at 12 weeks. Scale bars, 200 µm. (J‐L) Quantification of the percentage of affected area of Alcian blue, Sirius red, and AOD of Ki67 from KPC and KPSC pancreas (*n* = 3 mice per group) at 4, 8, and 12 weeks. *P*‐value by two‐way ANOVA test. (M) Kaplan‐Meier curve showing overall survival (OS) of KPC mice with or without SP1 knockout (*n* = 10 for KPSC, 40 for KPC). *P*‐value by log‐rank test. Data are shown as mean ± SD. *p < 0.05, **p < 0.01 and ***p < 0.001.

To explore the role of SP1 in PDAC tumorigenesis, we crossed *Sp1^LoxP/LoxP^* mice with *Kras^LSL‐G12D/+^; Trp53^LSL‐R172H/+^; Pdx1‐Cre* (KPC) mice, a classic genetic model for spontaneous PDAC development,^[^ ^30^ ^]^ to generate *Kras^LSL‐G12D/+^; Trp53^LSL‐R172H/+^; Sp1^LoxP/LoxP^; Pdx1‐Cre* (KPSC) mice (**Figure** **4**A). KPC and KPSC mice were sacrificed at 4, 8, and 12 weeks to compare tumor progression in the pancreas. Multi‐panel immunofluorescence staining was first performed to assess PDAC initiation and progression. Cytokeratin 19 (CK19) staining, a marker for lesions of ductal origin, revealed a marked decrease in the percentage of CK19‐positive areas in KPSC tissues compared to KPC tissues at 8 and 12 weeks (Figure 4B,C; Figure S4A, Supporting Information). Alpha‐smooth muscle actin (α‐SMA), used to identify cancer‐associated fibroblasts (CAFs) and pancreatic stellate cells (PSCs) in the stroma,^[^ ^31^ ^]^ also showed a marked reduction in α‐SMA‐stained areas in KPSC tissues at 12 weeks (Figure 4B,D; Figure S4A, Supporting Information). We also observed decreased expression of the mesenchymal marker Vimentin in KPSC tissues (Figure 4B,E; Figure S4A, Supporting Information), indicating reduced metastasis capability of KPSC tumor cells, as epithelial‐to‐mesenchymal transition (EMT) is a critical step in metastasis.^[^ ^32^ ^]^ Immunohistochemical staining was then used to analyze key histological markers of PDAC initiation and progression. Alcian blue staining revealed a significant decrease in sulfated/sialylated mucus in KPSC tissues compared to KPC tissues (Figure 4F,J; Figure S4B, Supporting Information). Ki67 staining indicated a significantly lower proliferation index in the KPSC pancreatic tissues than in KPC mice (Figure 4G,K). Sirius red staining showed that SP1 knockout significantly reduced collagen levels in the pancreas (Figure 4H,L). In terms of metabolism‐related enzymes, KPSC pancreatic tissues exhibited downregulated expression of HK2, PFKL, and LDHA, suggesting that SP1 deficiency inhibited glycolysis in these mice (Figure 4I). Lastly, SP1 knockout significantly prolonged the survival of KPC mice (Figure 4M). Taken together, the above results demonstrate the critical role of SP1 in PDAC tumorigenesis and tumor progression.

### 2.5 SP1 Regulates Aerobic Glycolysis in PDAC Cells

**Figure 5:**
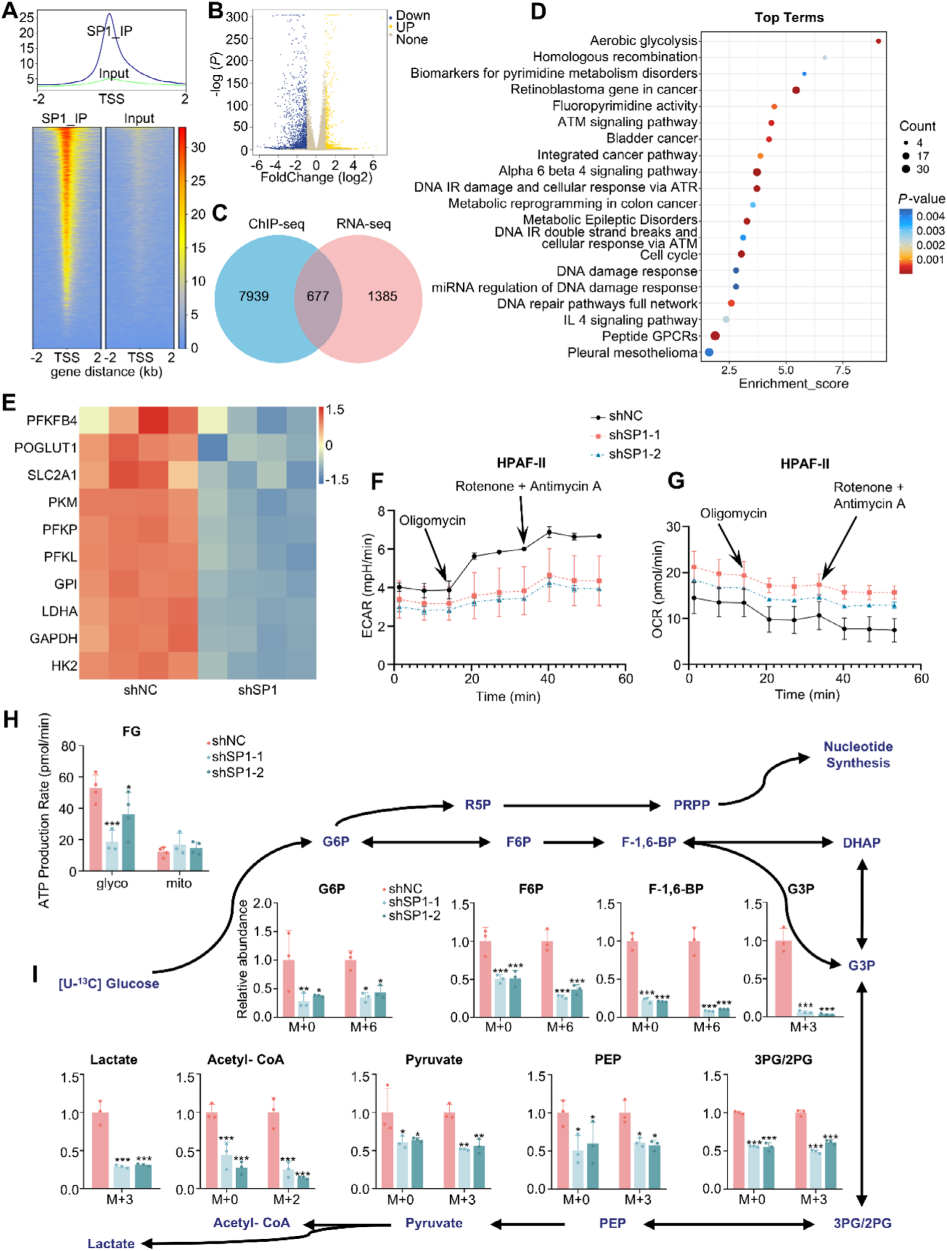
SP1 promotes aerobic glycolysis in PDAC cells by intensifying glycolytic flux. A) Identification of SP1 binding sites in FG cells by ChIP‐seq. B) Volcano plot of differentially expressed genes between control and SP1 knockdown FG cells by RNA‐seq. C) Venn diagram of differentially expressed genes in ChIP‐seq and RNA‐seq. D) Wikipathways enrichment analysis of differentially expressed genes in both ChIP‐seq and RNA‐seq. E) Heatmap of differentially expressed glycolytic enzymes in RNA‐seq. F, G) Seahorse assay results on ECAR (F) and OCR (G) of control and SP1 knockdown HPAF‐II cells. H) Seahorse assay results on ATP production rate of control and SP1 knockdown FG cells. *P*‐values by one‐way ANOVA test. I) Quantification of intermediary metabolites in the glycolysis pathway by isotope tracing in control and SP1 knockdown FG cells. *P*‐value by two‐way ANOVA test. Data are shown as mean ± SD. *p < 0.05, **p < 0.01 and ***p < 0.001.

To elucidate the mechanism by which SP1 regulates PDAC initiation and progression, we performed chromatin immunoprecipitation sequencing (ChIP‐seq) and RNA sequencing (RNA‐seq) on the FG PDAC cell line (**Figure** **5**A,B; Figure S5A, Supporting Information). By integrating the differentially expressed genes (DEGs) identified between control FG cells and cells with stable SP1 knockdown from both ChIP‐seq and RNA‐seq data, we selected 677 potential target genes of SP1 (Figure 5C). Wikipathways and KEGG pathway analyses revealed significant enrichment in glycolysis pathways (Figure 5D; Figure S5B, Supporting Information). A heatmap analysis further demonstrated that SP1 knockdown markedly reduced the expression of key glycolytic enzymes (Figure 5E).

To further explore the role of SP1 in glycolysis, we performed the Seahorse assay to measure the rate of adenosine triphosphate (ATP) production in both control and SP1‐knockdown PDAC cells. Knockdown of SP1 in FG or HPAF‐II cells significantly decreased the ATP production rate and extracellular acidification rate (ECAR), but had minimal effect on the oxygen consumption rate (OCR) of PDAC cells (Figure 5F–H; Figure S5C,D, Supporting Information), suggesting that SP1 positively regulates aerobic glycolysis in PDAC cells. We then performed a metabolic flux analysis on glycolytic activity and tricarboxylic acid (TCA) cycle in FG cells using ^13^C‐labeled glucose, pyruvate, and glutamine. The isotope tracing of aerobic glycolysis showed that the levels of several intermediary metabolites were reduced upon SP1 knockdown (Figure 5I). Besides, we observed an upregulation of several metabolites of the TCA cycle, such as citrate (M+2), succinate (M+2), fumarate (M+2), and malate (M+2), in SP1 knockdown cells (Figure S6, Supporting Information). This implies that SP1‐deficient PDAC cells may compensate by increasing the utilization of exogenously supplied pyruvate. Collectively, these data suggested that SP1 plays a crucial role in positively regulating aerobic glycolysis in PDAC cells.

### 2.6 SP1 Transcriptionally Activates PFKFB4 in PDAC cells

**Figure 6:**
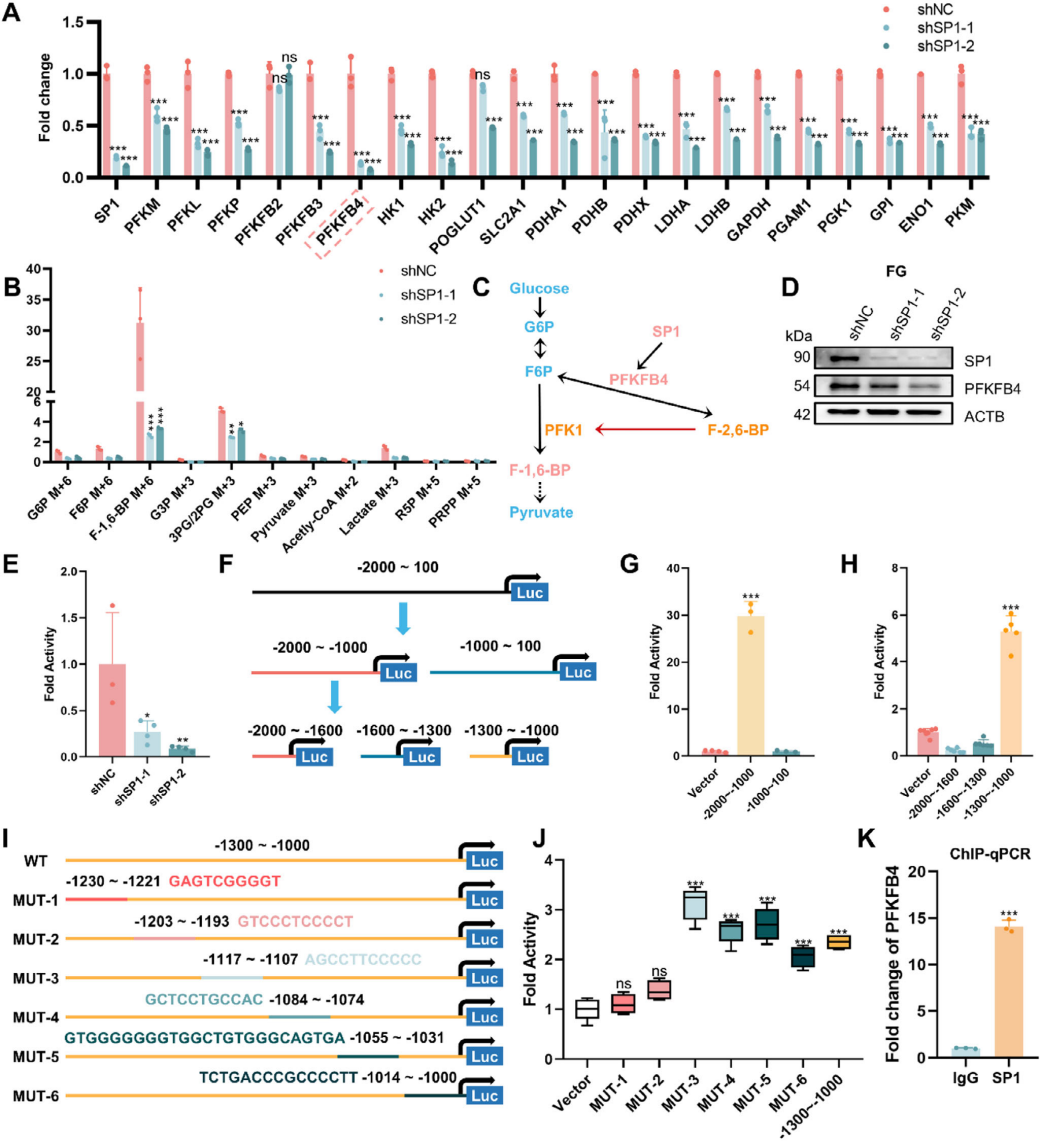
SP1 transcriptionally activates PFKFB4 by directly binding to its promoter. A) RT‐qPCR analysis of the expression of glycolysis‐related genes in control and SP1 knockdown FG cells. *P*‐value by two‐way ANOVA test. B) Relative expression of intermediary metabolites in the glycolysis pathway normalized to glucose 6‐phosphate (G6P). *P*‐value by two‐way ANOVA test. C) The proposed mechanism by which SP1 regulates aerobic glycolysis in pancreatic cancer cells. SP1 transcriptionally activated PFKFB4, which converts F6P to F‐2,6‐BP, which in turn activates the activity of PFK1, leading to the accumulation of F‐1,6‐BP in cancer cells. D) Effect of SP1 knockdown on PFKFB4 in FG cells. E) Dual‐luciferase reporter assay showing transcriptional activity of SP1 on *PFKFB4* promoter. *p*‐value by one‐way ANOVA test. F–H) Truncated dual‐luciferase reporter assay to narrow down the range of binding sites of SP1 on *PFKFB4* promoter. *p*‐value by one‐way ANOVA test. I, J) Dual‐luciferase reporter assay using mutated promoter sequences to pinpoint the binding sites of SP1 on the promoter of *PFKFB4*. *P*‐value by one‐way ANOVA test. K) ChIP‐qPCR assay confirmed the binding between SP1 and *PFKFB4* promoter. *P*‐value by Student's t‐test. Data are shown as mean ± SD. *p < 0.05, **p < 0.01 and ***p < 0.001.

To investigate the molecular mechanism by which SP1 modulates aerobic glycolysis in PDAC, we performed reverse‐transcription quantitative real‐time polymerase chain reaction (RT‐qPCR) assays on genes potentially involved in glycolysis. The results showed that SP1 knockdown in FG cells led to a decrease in the RNA levels of several glycolysis‐related genes (**Figure** **6**A), among which PFKFB4 (6‐phosphofructo‐2‐kinase/fructose‐2,6‐biphosphatase 4) showed the most significant downregulation. GO and InterPro analyses of the ChIP‐seq and RNA‐seq data revealed a strong correlation between SP1 and phosphofructokinase in PDAC (Figure S7A,B, Supporting Information). PFKFB4 is known to synthesize fructose‐2,6‐bisphosphate (F‐2,6‐BP) from fructose‐6‐phosphate (F6P), and F‐2,6‐BP acts as a potent stimulator of 6‐phosphofructokinase (PFK‐1), the key rate‐limiting enzyme of glycolysis that converts F6P to fructose‐1,6‐diphosphate (F‐1,6‐BP)^[^ ^33^ ^]^ (Figure 6C). Consistent with the role of PFKFB4 in glycolysis, quantification of glycolytic metabolites in control and SP1‐knockdown FG cells showed that F‐1,6‐BP was the most abundant intermediate metabolite, indicating upregulated PFK‐1 activity in PDAC cells; importantly, only F‐1,6‐BP showed significant downregulation after SP1 knockdown (Figure 6B). Thus, we hypothesized that SP1 regulates aerobic glycolysis in PDAC cells by modulating the expression of PFKFB4 and the catalytic activity of PFK‐1.

To test this hypothesis, we first examined the correlation between SP1 and PFKFB4 at the protein level. Immunoblotting experiments showed that the protein level of PFKFB4 decreased upon SP1 knockdown in FG cells (Figure 6D). A dual‐luciferase reporter assay further demonstrated that SP1 knockdown significantly reduced the transcriptional activity of PFKFB4 (Figure 6E). To identify the SP1 binding sites on the *PFKFB4* promoter, we truncated the promoter region of *PFKFB4* and utilized a dual‐luciferase reporter assay to narrow down the SP1 binding region to between −1300 nt and −1000 nt (Figure 6F–H). Next, we identified potential SP1 binding sites within this region by comparing its sequence with sequence motifs of conserved SP1 binding sites from the JASPAR database^[^ ^34^ ^]^ (Figure S7C, Supporting Information) and conducted mutational analysis on these sites in a dual‐luciferase reporter assay (Figure 6I). The results showed that mutations in the 3rd to 6th binding sites (MUT‐3–MUT‐6) did not affect the transcriptional activity promoted by SP1; however, mutations in the 1st and 2nd binding sites (MUT‐1 and MUT‐2) significantly decreased transcriptional activity (Figure 6J). This indicated that the SP1 binding sites on the *PFKFB4* promoter were located at −1230 to −1221 nt and −1203 to −1193 nt. Moreover, a ChIP‐qPCR assay in FG cells confirmed that SP1 directly binds to the *PFKFB4* promoter to activate its transcription (Figure 6K).

### 2.7 SP1 Regulate Aerobic Glycolysis in PDAC Cells through Activating PFKFB4

**Figure 7:**
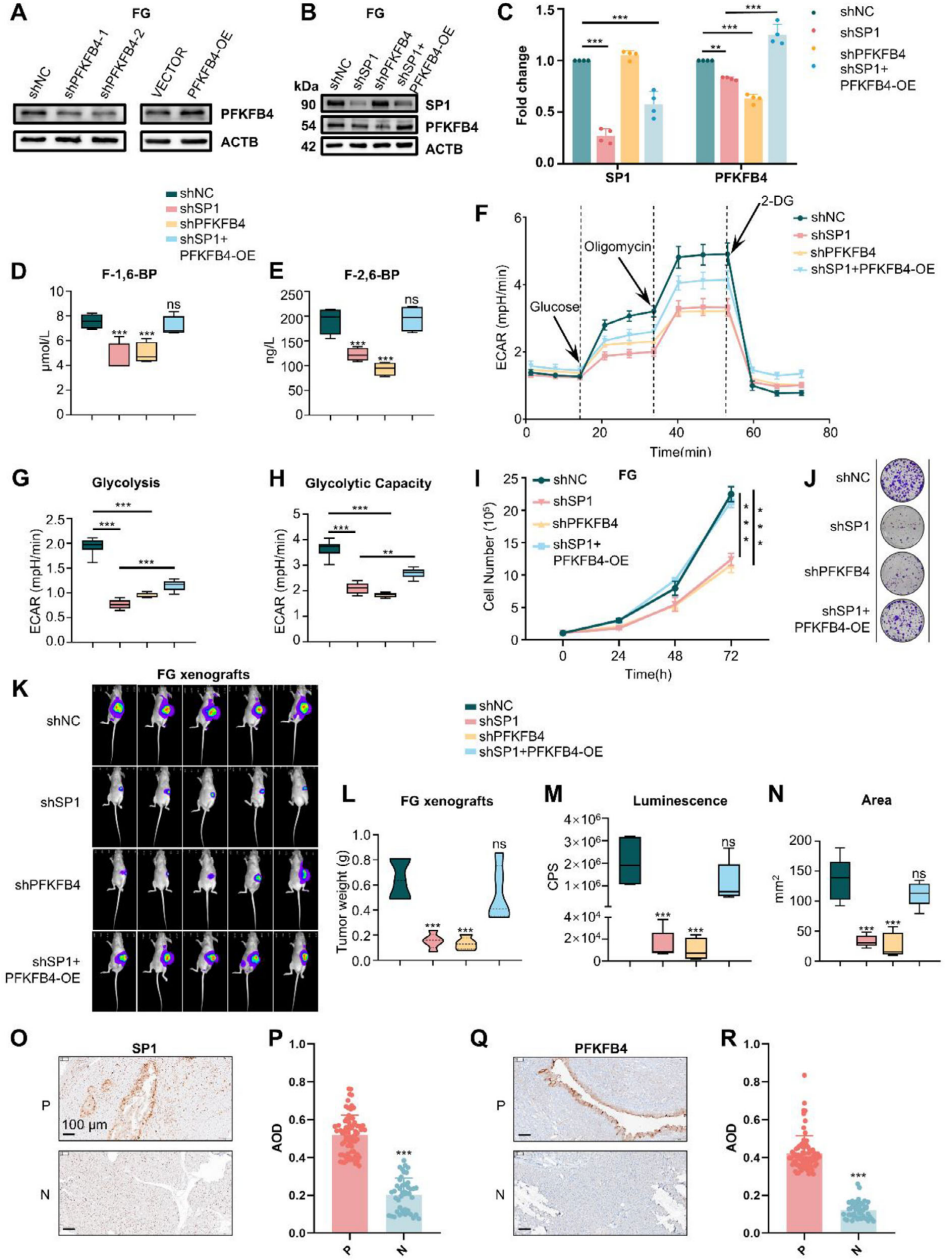
SP1 enhances aerobic glycolysis and progression of PDAC through transcriptional activation of PFKFB4. A–C) PFKFB4 overexpression rescued the decrease in PFKFB4 levels in FG cells as a result of SP1 or PFKFB4 knockdown. *P*‐value by two‐way ANOVA test. D, E) PFKFB4 overexpression rescued the decrease in F‐1,6‐BP and F‐2,6‐BP levels in FG cells as a result of SP1 or PFKFB4 knockdown. F‐1,6‐BP and F‐2,6‐BP levels were measured by ELISA. *P*‐value by one‐way ANOVA test. F–H) Seahorse assay showing that PFKFB4 overexpression rescued the decrease in aerobic glycolysis in FG cells as a result of SP1 or PFKFB4 knockdown. *P*‐value by one‐way ANOVA test. I, J) Proliferation (I) and colony formation (J) showing that PFKFB4 overexpression rescued the growth suppression in FG cells induced by SP1 or PFKFB4 knockdown in vitro. *P*‐value by two‐way ANOVA test. K–N) PFKFB4 overexpression rescued the growth suppression of FG xenograft tumors induced by SP1 or PFKFB4 knockdown in vivo. (K) In vivo imaging of FG xenograft tumors (*n* = 5 mice per group). (L) Quantification of tumor weight. *P*‐value by one‐way ANOVA test. (M, N) Quantification of the IVIS signal in K. O, P) Representative images and AOD of SP1 staining of human PanIN (*n* = 75) and normal (*n* = 45) tissues. Q, R) Representative images and AOD of SP1 staining of human PanIN (*n* = 75) and normal (*n* = 45) tissues. Scale bars, 100 µm. *p*‐value by Unpaired Student's t‐test. Data are shown as mean ± SD. **p < 0.01 and ***p < 0.001.

To determine whether PFKFB4 activation is required for SP1‐mediated aerobic glycolysis in PDAC cells, we used lentiviral vectors to knock down or overexpress PFKFB4 in FG cells (**Figure** **7**A). While PFKFB4 knockdown had no effect on SP1 levels, PFKFB4 overexpression (OE) effectively restored PFKFB4 expression that was reduced by SP1 knockdown (Figure 7A–C). Metabolite analysis revealed that both SP1 knockdown and PFKFB4 knockdown markedly decreased the levels of F‐1,6‐BP and F‐2,6‐BP in PDAC cells, and PFKFB4 OE reversed the effects of SP1 knockdown (Figure 7D,E). Using the Seahorse system, we observed that both SP1 knockdown and PFKFB4 knockdown significantly inhibited glycolysis in PDAC cells, while PFKFB4 overexpression partially restored glycolysis in SP1‐knockdown cells (Figure 7F–H). In vitro experiments showed that both SP1 and PFKFB4 knockdown significantly suppressed the proliferation and colony formation of FG cells, and PFKFB4 overexpression rescued the growth suppression caused by SP1 silencing (Figure 7I,J). In vivo experiments in CDX models of luciferase‐labeled FG cells revealed that SP1 and PFKFB4 knockdown also suppressed tumor growth, and the inhibitory effect of SP1 knockdown on tumor growth was rescued by PFKFB4 overexpression (Figure 7K–N; Figure S8, Supporting Information). We further validated the expression of SP1 and PFKFB4 in a clinical cohort composed of 75 PanIN and 45 normal pancreas tissues by IHC staining and observed significant upregulation of SP1 and PFKFB4 in PanIN lesions (Figure 7O–R).

**Figure 8:**
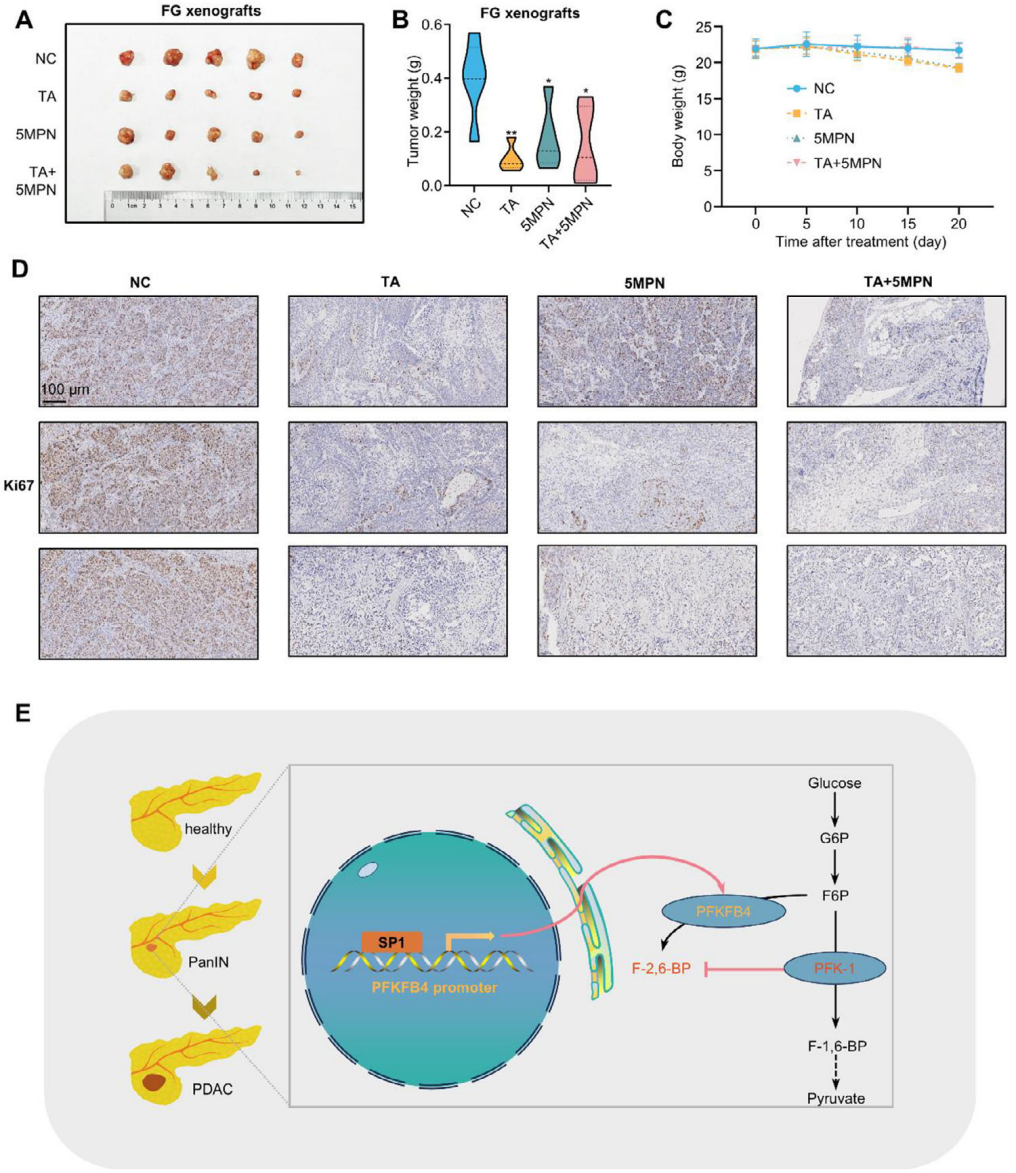
Pharmacological inhibition of SP1 and PFKFB4 restrains PDAC growth. A–D) Effect of SP1 and PFKFB4 inhibitors on the growth of FG cells in vivo. FG cells were orthotopically implanted onto the pancreas of nude mice. Mice were then treated with TA (gavage, 80 mg kg^−1^, every 2 days), 5MPN (gavage, 120 mg kg^−1^, every 2 days), or a combination of TA (40 mg kg^−1^) and 5MPN (60 mg kg^−1^). (A) Image of FG tumors 20 days after treatment. (B) Tumor weight at the end of the experiment (*n* = 5 mice per group). *p*‐value by one‐way ANOVA test. (C) Body weight of tumor‐bearing nude mice over the course of drug treatment. *p*‐value by two‐way ANOVA test. (D) Representative images of Ki67 staining of FG CDX tumors treated with TA, 5MPN, or a combination of TA and 5MPN. Scale bars, 100 µm. (E) Schematic diagram showing that SP1 binds to the promoter of *PFKFB4* to activate its transcription, which increases the activity of PFK‐1 and glycolytic flux and promotes the tumorigenesis and progression of PDAC. Data are shown as mean ± SD. *p < 0.05 and **p < 0.01.

Lastly, to explore the therapeutic potential of targeting SP1 and PFKFB4 in PDAC, we orthotopically transplanted FG cells into nude mice and treated them with TA (an SP1 inhibitor), 5MPN^[^ ^35^ ^]^ (a selective inhibitor of PFKFB4), or a combination of TA and 5MPN at half doses. TA, 5MPN, and the combination treatment all inhibited tumor growth without significantly affecting the body weight of the mice (**Figure** **8**A–D). In conclusion, SP1 enhances aerobic glycolysis and proliferation in PDAC cells by transcriptionally activating PFKFB4 (Figure 8E).

## 3 Discussion

The early diagnosis of pancreatic cancer remains a prevailing clinical challenge, with no consensus on an effective screening tool for early‐stage detection. The most widely used biomarker carbohydrate antigen 19‐9 (CA19‐9) bears certain predictive value for late‐stage PDAC and is useful for monitoring treatment response,^[^ ^36^ ^]^ however, it is not sensitive and specific for early detection.^[^ ^37^ ^]^ Despite extensive efforts to identify novel biomarkers and therapeutic targets for the early diagnosis and treatment of PDAC—leveraging cutting‐edge technologies such as serum lipidomics, exosome profiling, circulating tumor DNA analysis, circulating tumor cell sequencing, and whole‐exosome sequencing of driver mutations^[^ ^38^, ^39^, ^40^, ^41^, ^42^, ^43^ ^]^—only a few genetic mutations, such as those in *KRAS*, *TP53*, *CDKN2A*, and *SMAD4*, are consistently implicated in driving PDAC initiation and progression.^[^ ^44^, ^45^, ^46^, ^47^ ^]^ Moreover, most studies to date have relied on bulk tumor tissues from PDAC patients, which contain a mixture of tumor cells and adjacent “normal” cells, making it difficult to pinpoint factors specific to early‐stage precursor lesions or to dissect the distinct processes of tumor initiation (before PanIN) and progression (from PanIN to PDAC) separately.

Herein, we addressed these challenges by isolating pure PanIN tissues using LCM and analyzing the proteomic changes within the PanIN tissue. Two key assumptions underpinned this approach: 1) proteins in paraffin‐embedded tissues remain stable over time, and 2) the PanIN lesions identified in advanced‐stage PDAC patients reflect early‐stage PDAC initiation and progression within the same patient. Our proteomic analysis indicated that significant metabolic remodeling already occurred at the PanIN stage, suggesting that metabolic remodeling may be a driver of tumorigenesis rather than an adaptation to the tumor microenvironment in advanced PDAC.

Previous studies have demonstrated that mutant *KRAS* can reprogram metabolism in PDAC to facilitate tumor adaptation to a harsh microenvironment.^[^ ^12^, ^19^, ^48^ ^]^ However, other molecular regulators contributing to this metabolic remodeling remain poorly characterized. In this study, we identified SP1 as a novel regulator of glycolysis in PDAC. We found that: 1) SP1 is overexpressed in both PanIN and PDAC tissues; 2) SP1 promotes tumorigenesis in the KPC model; 3) SP1 promotes tumor growth in PDAC cell lines and patient‐derived organoids. Hence, SP1 drives both tumor initiation and progression. By integrating ChIP‐seq data with metabolic flux assays, we demonstrated that SP1 drives aerobic glycolysis in PanIN and PDAC through the transcriptional activation of PFKFB4. Our findings reveal that SP1‐driven glycolytic flux facilitates PDAC initiation and progression through dual bioenergetic and biosynthetic mechanisms: 1) ATP hyperproduction: PFKFB4, transcriptionally activated by SP1, increases F‐2,6‐BP allosteric activation of PFK‐1, accelerating glycolytic ATP production to meet elevated energy demands of survival and proliferation due to the oligotrophic and highly hypoxic PDAC microenvironment, created by hypovascularization and the desmoplastic reaction;^[^ ^49^ ^]^ 2) Anabolic precursor supply: Glycolytic intermediates serve as substrates for the pentose phosphate pathway (PPP), generating nicotinamide adenine dinucleotide phosphate (NADPH) for redox homeostasis and nucleotides for DNA replication.^[^ ^12^, ^50^ ^]^ Consistently, SP1 knockdown reduced F‐1,6‐BP abundance – a node branching to PPP – explaining suppressed cell growth and colony formation. Thus, metabolic remodeling in PanIN lesions represents an early adaptive strategy to support biomass accumulation prior to angiogenesis and colonization, potentially collaborating with late‐stage metabolic adaptations that cope with hypoxia.^[^ ^51^ ^]^ Correspondingly, pharmacological inhibition or genetic knockdown of SP1 or PFKFB4 effectively reversed aerobic glycolysis and suppressed tumor growth, demonstrating the potential therapeutic value of targeting these molecular targets.

Beyond its metabolic regulation functions, SP1 may serve as an integrator of inflammatory signaling cascades, thereby contributing to tumorigenesis in PDAC. According to reports, protein kinase C delta (PKCδ) is upregulated in chronic pancreatitis, a well‐established risk factor for PDAC.^[^ ^8^ ^]^ And the overexpression of PKCδ correlates with acinar‐to‐ductal metaplasia (ADM),^[^ ^52^, ^53^ ^]^ the initial histopathological alteration in pancreatic carcinogenesis.^[^ ^54^, ^55^ ^]^ Critically, PKCδ phosphorylates SP1 to enhance its DNA‐binding affinity, and SP1 reciprocally regulates *PKCδ* in response to insulin signaling,^[^ ^56^ ^]^ establishing a positive feedforward loop. Supporting this, inhibition of SP1 reduces pain sensitivity and inflammatory cytokines (like interleukin‐6, tumor necrosis factor‐α, and so on) in chronic pancreatitis models,^[^ ^57^ ^]^ These observations display a coherent pathogenic cascade: inflammation – SP1 activation – metabolic remodeling – PanIN progression. Therapeutically, co‐targeting SP1 and its downstream effector PFKFB4 disrupts this cascade, and their synergy in vivo underscores the axis's efficacy. We suppose that combining anti‐inflammatory agents with SP1‐PFKFB4 inhibition may prevent inflammation‐driven SP1 activation, offering a novel preventive strategy for high‐risk individuals.

There are still several limitations during the implementation of this study, which need to be further improved and enhanced in the future. We acknowledge that the proteomic analysis of five PanIN lesions may not fully represent the broader spectrum of these lesions. Further validation of our conclusions is needed using a larger and more diverse set of clinical samples. Additionally, due to the lack of suitable in vitro models for PanIN, we were limited to using PDAC cell lines for most experiments, which may not fully recapitulate the biological processes underlying PDAC initiation. Moreover, given the absence of widely accepted biomarkers for PanIN, isolating live PanIN‐derived cells from patients or KPC mice remains challenging. Genomic analyses confirm that PDAC cells retain identical driver mutations to those in adjacent PanINs,^[^ ^45^ ^]^ suggesting conserved oncogenic dependencies. Corroborating this genetic continuity, SP1 upregulation was observed in both human PanINs and invasive PDACs, suggesting preservation of this regulatory axis during malignant progression. Furthermore, we employed KPC mice to mimic PanIN‐to‐PDAC progression, wherein genetic ablation of SP1 suppressed tumor development. Besides, our core finding – metabolic remodeling in PanIN – is grounded in direct analysis of clinical specimens isolated by LCM, independent of cell lines. Nevertheless, the absence of pure PanIN in vitro models remains a non‐negligible problem. Future efforts should focus on gaining more detailed insights into PanIN progression, particularly through spatial transcriptomics and single‐cell sequencing approaches. We are trying to establish long‐term expandable PanIN organoids from *Kras^LSL‐G12D/+^; Pdx1‐Cre* (KC) mice, and future studies will compare SP1‐PFKFB4 axis function across PanIN organoids and PDAC cell lines, resolving stage‐specific adaptations. Also, as a ubiquitous transcription factor, SP1 regulates diverse biological processes, which raises legitimate concerns about off‐target effects. To alleviate such effects, we designed two distinct shRNA lentiviruses to genetically knock down SP1 and utilized two inhibitors of SP1 (TA and MIT) with complementary pharmacological mechanisms. Conditional knockout of *Sp1* in KPC mice significantly attenuated tumorigenesis, supporting its biological function in vivo. PFKFB4 overexpression reversed glycolytic suppression and growth inhibition in SP1‐knockdown cells, indicating the specificity of the SP1‐PFKFB4 axis in glycolytic flux. Combined SP1/PFKFB4 inhibition demonstrated synergy in tumor suppression without observable toxicity in mice at effective doses.

## 4 Conclusion

In conclusion, our study identified a critical role of SP1 in driving aerobic glycolysis during the initiation and progression of PDAC. Targeting SP1 or its downstream effector PFKFB4 effectively inhibits tumorigenesis and tumor growth in both mouse models and patient‐derived organoid models of pancreatic cancer. These findings suggest that SP1 upregulation and associated metabolic remodeling are key biomarkers of early‐stage PDAC, offering new avenues for the early diagnosis, prevention, and treatment of this highly aggressive cancer.

## 5 Experimental Section

### Human PDAC Specimens

All human cancer tissues were obtained from the Department of Pancreatic and Metabolic Surgery, Nanjing Drum Tower Hospital, Affiliated Hospital of Medical School, Nanjing University, Nanjing, China. Tissues were obtained with patients’ written consent under a protocol (2023‐656‐01) approved by the Institutional Review Board and confirmed by a pathologist before use.

### Cell Lines and Culture

Human pancreatic cancer cell lines PANC‐1 (RRID: CVCL_0480), MIA PaCa‐2 (RRID: CVCL_0428), HPAC (RRID: CVCL_3517), FG (RRID: CVCL_8196), BxPC‐3 (RRID: CVCL_0186), Capan‐1 (RRID: CVCL_0237), Capan‐2 (RRID: CVCL_0026), CFPAC‐1 (RRID: CVCL_1119), PaTu 8902 (RRID: CVCL_1845), PSN1 (RRID: CVCL_1644), HPAF‐II (RRID: CVCL_0313), Panc 05.04 (RRID: CVCL_1637) and S2‐013 (RRID: CVCL_B280) were obtained from American Type Culture Collection (ATCC) or kindly provided by Dr. Chao Yan (Nanjing University, Nanjing, China). Cells were cultured in Dulbecco's modified Eagle's medium (DMEM) or Roswell Park Memorial Institute (RPMI) or Iscove's Modified Dulbecco's Medium (IMDM) with 10%–20% fetal bovine serum (FBS) at 37 °C atmosphere with 5% CO2 according to ATCC instructions. All media were supplemented with penicillin (100 mg mL^−1^) and streptomycin (100 mg/mL). Cells were regularly checked for bacterial, fungal and mycoplasma contamination, and the cells used for analysis were contamination‐free. FG^[^ ^58^, ^59^, ^60^ ^]^ and HPAF‐II^[^ ^61^, ^62^ ^]^ have been previously validated and described, and they were chosen for further exploration in this study by immunoblot.

### Lentivirus Construction

Lentivirus vectors for knocking down SP1 and PFKFB4 and overexpressing PFKFB4 were obtained from Genechem (Shanghai, China). The following shRNA sequences were used. SP1‐1: GCTGGTGGTGATGGAATACAT; SP1‐2: TGGCAGTGAGTCTTCCAAG; PFKFB4‐1: GACGTGGTCAAGACCTACAAA; PFKFB4‐2: GCTGGCCTACTTCCTCGACAA.

### Cell Proliferation Assay

PDAC cells (1 × 10^5^ cells/well) were seeded into each well of 6‐well plates in triplicate and allowed to grow for three days. Cell numbers were determined by cell counting with a hemocytometer at 0, 24, 48, and 72 h after seeding.

### Colony Formation Assay

PDAC cells (500 cells per well) were seeded into 6‐well plates and incubated with complete medium for 2 weeks. The colonies were fixed with 4% paraformaldehyde and stained with 0.1% crystal violet. Images were then taken under a stereoscopic microscope and quantified using ImageJ.

### Western Blotting

Total protein lysates were extracted using Radio Immunoprecipitation Assay (RIPA) lysis buffer (89901, Thermo Fisher) with protease inhibitor cocktail (HY‐K0010, MCE) on ice. The protein concentration was determined using a bicinchoninic acid (BCA) protein quantification kit (E112, Vazyme). The proteins were then separated on a 10% sodium dodecyl sulfate – polyacrylamide gel electrophoresis (SDS‐PAGE) gel and transferred onto polyvinylidene difluoride (PVDF) membranes with a pore size of 0.22 µm, which was blocked with 5% bovine serum albumin (BSA) in Tris‐Buffered Saline with Tween 20 (TBST) for 1 h at room temperature. The membranes were then incubated with specific primary antibodies, including β‐actin (AC026, ABclonal), VEGFA (A5708, ABclonal), SP1 (9389, CST), PFKFB4 (ab137785, Abcam) overnight at 4 °C. Afterward, the membranes were incubated with horseradish peroxidase (HRP)‐conjugated secondary antibodies (111‐035‐003, Jackson Laboratory) for 1 h at room temperature. The proteins of interest were then examined using the enhanced chemiluminescence (ECL) kit (FD8030, FDbio) and quantified using Image J.

### RNA Isolation and Real‐Time Quantitative PCR

The total RNA was extracted with RNA Isolator Total RNA Extraction Reagent (R401, Vazyme), and the concentration of RNA was detected with NanoDrop. The RNA was reverse transcribed to cDNA using a HiScript RT SuperMix kit (R122‐01, Vazyme). Then, quantitative real‐time polymerase chain reaction (qPCR) was performed using the SYBR qPCR Kit (Q711‐02, Vazyme). The Cycle Threshold (CT) value of ACTB was used as the internal reference, and the relative RNA expression of targeted genes was analyzed using the ΔΔCt method. The primer sequences used are listed in Table S1 (Supporting Information).

### Chromatin Immunoprecipitation PCR (ChIP)

ChIP assay in FG cells was performed using a ChIP assay kit (26157, Thermo Fisher) following the manufacturer's instructions. The immunoprecipitation step was performed using an antibody against SP1 (9389, CST). The primers used for the PCR step were PFKFB4‐F: CAGATGGCTGACGACCGTTT and PFKFB4‐R: GAGCCACAATGGGGGTTTGT.

### Dual‐Luciferase Reporter Assay

The dual‐luciferase reporter plasmids containing the full‐length *PFKFB4* promoter sequence, the truncated *PFKFB4* promoter sequences, and the mutated *PFKFB4* promoter sequences were respectively constructed and transfected into FG cells. The pRL‐TK Renilla luciferase reporter plasmid vector was used for internal normalization. A Dual Luciferase Reporter Assay Kit (DL101‐01, Vazyme) was used to measure the firefly and Renilla luciferase activity following the manufacturer's instructions.

### RNA‐seq and ChIP‐seq

For RNA‐seq, total RNA was extracted as described above and treated with Oligo (dT) labeled magnetic beads to enrich and purify mature mRNA. A fragmentation buffer was then added to fragment the mRNAs. These fragments were reverse transcribed into cDNA with random primers. The cDNA fragments were purified using the QIAQuick PCR kit (28104, QIAGEN), end‐repaired, poly(A) added, and ligated to Illumina sequencing adapters. The fragments within the right target size were recovered by agarose gel electrophoresis, amplified by PCR, and sequenced using Illumina HiSeq2500 by GENOME.

For ChIP‐seq, the DNA fragments that can bind to SP1 were enriched and extracted with a ChIP assay kit (26157, Thermo Fisher) using an SP1 antibody (9389, CST). The pulled‐down DNA was end‐repaired, poly(A) added, and ligated to Illumina sequencing adapters. The fragments within the right target size were recovered by magnetic beads and amplified by PCR. The constructed library was then inspected by agarose gel electrophoresis and sequenced by LC‐bio Co.

### Mouse Strain

The *Kras^LSL‐G12D/+^; Trp53^LSL‐R172H/+^* (KP) mice, *Pdx1‐Cre* mice, and floxed Sp1 (*Sp1^LoxP/LoxP^*) mice were purchased from Gempharmatech Co., Ltd (Nanjing, China). The above mice were interbred to generate *Kras^LSL‐G12D/+^; Trp53^LSL‐R172H/+^; Sp1^LoxP/LoxP^; Pdx1‐Cre* (KPSC) mice and *Kras^LSL‐G12D/+^; Trp53^LSL‐R172H/+^; Pdx1‐Cre* (KPC) mice. Mice were genotyped by PCR. NOD/ShiLtJGpt‐Prkdc^em26Cd52^Il2rg^em26Cd22^/Gpt (NCG) mice and BALB/cNj‐Foxn1^nu^/Gpt (nude) mice at 5–6 weeks were purchased from GemPharmatech Co., Ltd (Nanjing, China). Only female mice were used for further analysis. All mice were housed in a Specific Pathogen Free (SPF) facility with free access to food and water. Animal experiments were performed under a protocol (2023AE01039) approved by the Laboratory Animal Ethics Committee of Nanjing Drum Tower Hospital.

### Human PDAC Organoids and Xenografts in Mice

PDAC PDOs were established according to an existing protocol,^[^ ^63^ ^]^ For PDO xenografts (PDOX), organoids were dissociated into a single cell suspension, and 1 × 10^6^ cells in 50 µL phosphate‐buffered saline (PBS) were injected orthotopically into the pancreas of NCG mice. Mice bearing PDAC PDO xenografts were randomly assigned to three groups: NC (nontreated control), TA (tolfenamic acid, gavage, 80 mg kg^−1^, every 3 days), and MIT (mithramycin A, intraperitoneal injection, 1.5 mg kg^−1^, every 3 days). Tumor‐bearing mice were sacrificed 4 weeks after treatment, and tumor weight was measured.

For xenograft growth studies using PDAC cell lines, 1 × 10^6^ control or genetically‐modified FG or HPAF‐II cells (in 50 µL PBS) were injected orthotopically into the pancreas of nude mice. In drug‐treatment experiments, nude mice bearing FG xenografts were randomly assigned to four groups: control, TA (gavage, 80 mg kg^−1^, every 2 days), 5MPN (gavage, 120 mg kg^−1^, every 2 days), or a combination of TA (40 mg kg^−1^) and 5MPN (60 mg kg^−1^). Mice were sacrificed 20 days after treatment, and tumors were evaluated.

### Laser Capture Microdissection (LCM)

Hematoxylin‐eosin (HE) stained sections of formalin‐fixed paraffin‐embedded PDAC tissues were evaluated by a pathologist, and five cases containing high‐grade PanINs were selected. 5 µm tissue sections were cut and placed on a Polyethylene naphthalate two formic acid glycol ester (PEN) membrane glass slide. Target areas that were designated as PanIN were isolated as descibed,^[^ ^5^, ^13^ ^]^ aided by Shanghai Freedom Bio‐Tech Co., Ltd.

### LC‐MS/MS Analysis—High Performance Liquid Chromatography (HPLC) Analysis

The separation was performed on an 1100 HPLC System (Agilent) using a Nano Chrom‐C18 column (5 µm, 150 × 2.1 mm). Mobile phases A (2% acetonitrile in HPLC water) and B (90% acetonitrile in HPLC water) were used for the gradient. The solvent gradient was set as follows: 0–10 min, 98% A; 10–10.01 min, 98%–95% A; 10.01–37 min, 95%–80% A; 37–48 min, 80%–60% A; 48–48.01 min, 60%–10% A; 48.01–58 min, 10% A; 58–58.01 min, 10%–98% A; 58.01–63 min, 98% A. Tryptic peptides were separated at a fluent flow rate of 250 µL min^−1^ and monitored at 210 nm. Samples were collected for 10–50 min, and eluent was collected in centrifugal tubes 1–10 every minute in turn. Samples were recycled in this order until the end of the gradient. The separated peptides were lyophilized for mass spectrometry.

### LC‐MS/MS Analysis—Mass Spectrometry (MS) Analysis

Nanoflow reversed‐phase chromatography was performed on a nanoElute liquid chromatography system (Bruker Daltonics). Peptides were separated in 90 min at a flow rate of 300 nL min^−1^ on a 25 cm × 75 µm column (1.6 µm C18, ionopticks). Mobile phases A and B were 0.1 vol% formic acid solution and 80: 20: 0.1 vol% ACN: water: formic acid, respectively. The total run was 60 min (0–45 min, 22% B;45–50 min, 22%–37% B;50–55 min, 37%–80% B;55–60 min, 80% B).

### LC‐MS/MS Analysis—Data‐Independent Acquisition (DIA)

To perform DIA, we used the instrument control software (Bruker otofControl v6) to define quadrupole isolation windows as a function of the TIMS scan time (diaPASEF). Capillary voltage was 1.4 kV, dry gas temperature was 180 °C, and dry gas flow rate was 3.0 L min^−1^. The full MS scan range was set from 100 to 1700 m z^−1^. The collision energy was ramped linearly as a function of the mobility from 59 eV at 1/K0 = 1.6Vs cm^−2^ to 20 eV at 1/K0 = 0.7Vs cm^−2^.

### LC‐MS/MS Analysis—Database Search

Spectronaut (Version 15.3.210906.50606) was used to search all of the raw data thoroughly against the sample protein database. A database search was performed with Trypsin digestion specificity. Alkylation on cysteine was considered a fixed modification. Protein, peptide, and PSM's false discovery rate (FDR) were all set to 0.01. For DIA data, the quantification FDR was also set to 0.05. The quantity MS‐level was set at MS2.

### Immunohistochemistry (IHC) and Multi‐Panel Immunofluorescence (mIF)

IHC: FFPE tissue slides were deparaffinized and then rehydrated through an ethanol series, followed by antigen retrieval with sodium citrate buffer. Slides were blocked with 5% normal goat serum with 0.1% Triton X‐100 and 3% H_2_O_2_ in TBST for 1 h at room temperature and then incubated with primary antibodies (SP1: 9389, CST; PFKFB4: ab137785, Abcam; Ki67: ab15580, Abcam; HK2: ab209847, Abcam; PFKL: A23687, ABclonal; LDHA: 3582, CST; GLS: A11043, ABclonal; GLUD1/2: A21792, ABclonal; CD36: ab133625, Abcam; ACSL4: ab155282, Abcam; CPT2: A12426, ABclonal) overnight at 4 °C. Expression levels of those antigens were then detected by HRP‐conjugated diaminobenzidine (DAB).

### Multi‐Panel Immunofluorescence with Tyramide Signal Amplification (TSA)

FFPE tissue slides were deparaffinized and rehydrated. Slides were washed with deionized water followed by antigen retrieval at 96 °C with 10 mmol L^−1^ sodium citrate buffer (pH 6.0). Endogenous peroxides were quenched with 3% H_2_O_2,_ blocked with 3% BSA. Slides were incubated with primary antibodies (CK19: ab52625, Abcam; α‐SMA: MA1‐06110, Invitrogen; Vimentin: 5741, CST) overnight at 4 °C. Slides were washed three times with TBST and incubated in secondary antibody for 1 h. TSA conjugated to fluorophores was applied for 10 min and incubated with the reaction stop solution. At last, the slides were incubated with DAPI and sealed.

### Sankey Diagram of Transcription Factors (TF) and Metabolic Enzymes

We picked out all the differentially expressed TFs and metabolic enzymes in our PanIN proteomic profile and verified the regulatory relationships among them using the Transcriptional Regulatory Relationships Unraveled by Sentence‐based Text Mining (TRRUST) tool. Bioinformatic analysis was performed using the OECloud tools at https://cloud.oebiotech.com.

### Statistical Analysis

Statistical analyses were performed using GraphPad Prism 8 (GraphPad Software, Inc., La Jolla, CA). The statistical tests performed were paired or unpaired Student's t‐test, one‐way analysis of variance (ANOVA), two‐way ANOVA, Spearman test, or log‐rank analysis. Before applying parametric tests, data normality was assessed using the Shapiro‐Wilk test, and homogeneity of variances was evaluated using Levene's test. Data were presented as mean ± standard deviation (SD). Significance was set as *p < 0.05, **p < 0.01, ***p < 0.001, and ns: no significance for all data.

### Data Availability

Omics data URL: https://www.iprox.cn/page/PSV023.html;?url = 17271733998422 (Proteomics); https://dataview.ncbi.nlm.nih.gov/object/PRJNA1164179?reviewer = vtdlmdf5a37qs3t8lhdo2sjrll (RNA‐seq); https://dataview.ncbi.nlm.nih.gov/object/PRJNA1164409?reviewer = gbrj8q4fikf9s1gp4at697e11o (ChIP‐seq).

## Conflict of Interest

The authors declare no conflict of interest.

## Author Contributions

H.H., M.Y., L.Z., and N.T. contributed equally to this work. H.H. wrote, reviewed, and edited the original draft, performed methodology, visualization, investigation, data curation, and formal analysis. M.Y. performed validation and data curation. L.Z. and Z.C. performed formal analysis and data curation. N.T. and Y.C. performed methodology and data curation. X.F. performed the methodology and resources. M.Y., L.Y., J.W., J.T., Q.L., and X.F. performed the methodology. Y.X. performed Data curation. L.M. and B.K. performed supervision. J.C., F.M., and X.H. performed resources. C.Y. performed supervision and funding acquisition. Y.Q. performed supervision, resources, funding acquisition, wrote, reviewed, and edited the original draft. H.C. wrote, reviewed, and edited the original draft, performed supervision, formal analysis, and project administration.

## Supporting information



Supporting Information

## Data Availability

The data that support the findings of this study are available on request from the corresponding author. The data are not publicly available due to privacy or ethical restrictions.
