# Supplementary material for: SP1‐Mediated Glycolytic Reprogramming Promotes Tumorigenesis and Progression in Pancreatic Cancer
Source: Adv Sci (Weinh). 2025 Aug 20;12(42):e10071. doi: 10.1002/advs.202510071 (PMC12622429; doi:10.1002/advs.202510071)
Supplement: Supplementary file 1 — Supporting Information [file ADVS-12-e10071-s001.docx]

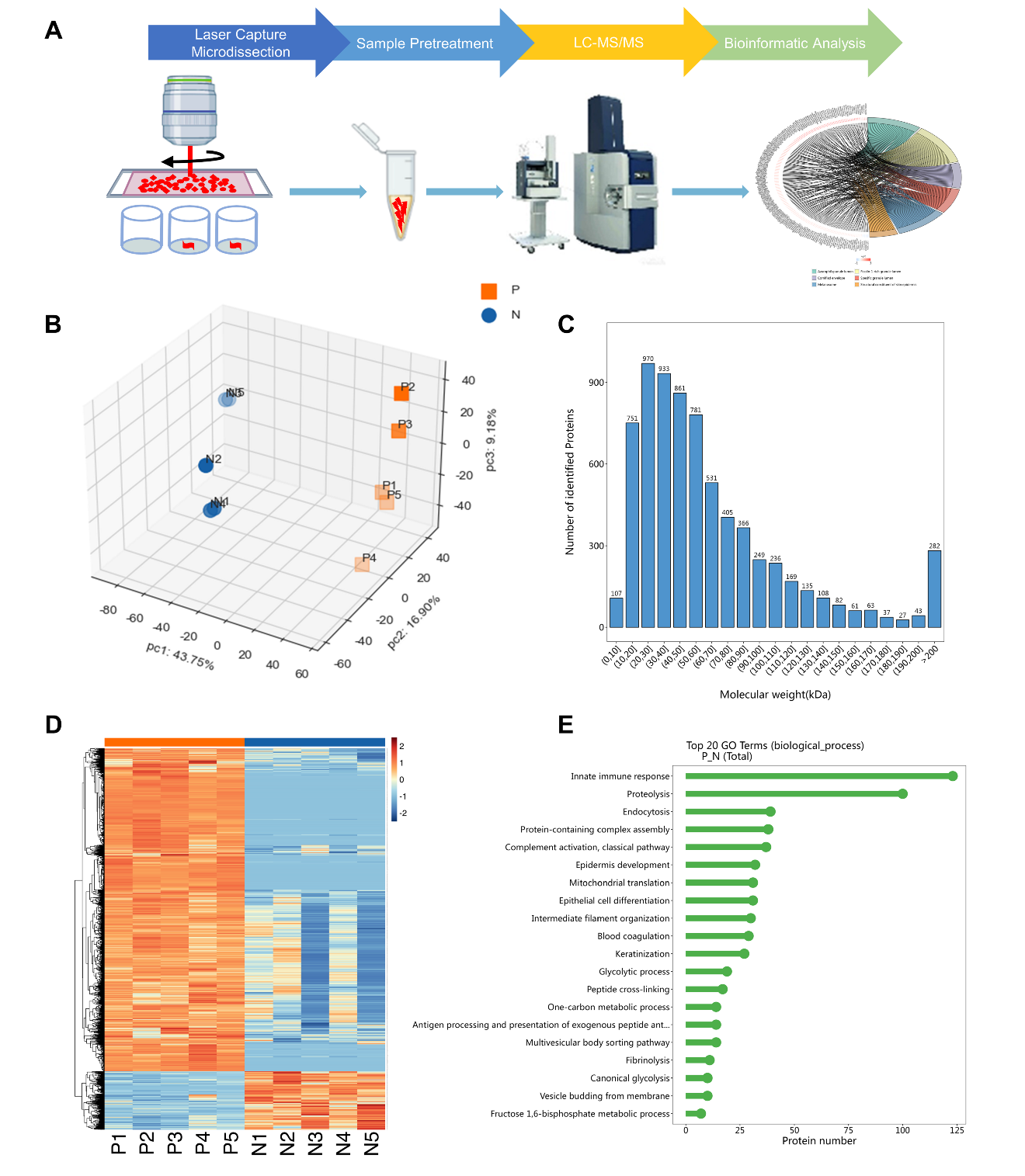


**Supplementary Figure 1. LCM-based proteomics of PanIN and normal pancreatic tissues.**

(A) Flow diagram of sample preparation and sample processing. (B) Principal component analysis (PCA) of the proteomic data showing clustering of PanIN tissues and normal tissues. (C) Distribution of the number of detected proteins over molecular masses. (D) Heatmap of differentially expressed proteins between PanIN tissues and normal tissues. (E) Top 20 terms of Gene Ontology (GO) biological process annotations.


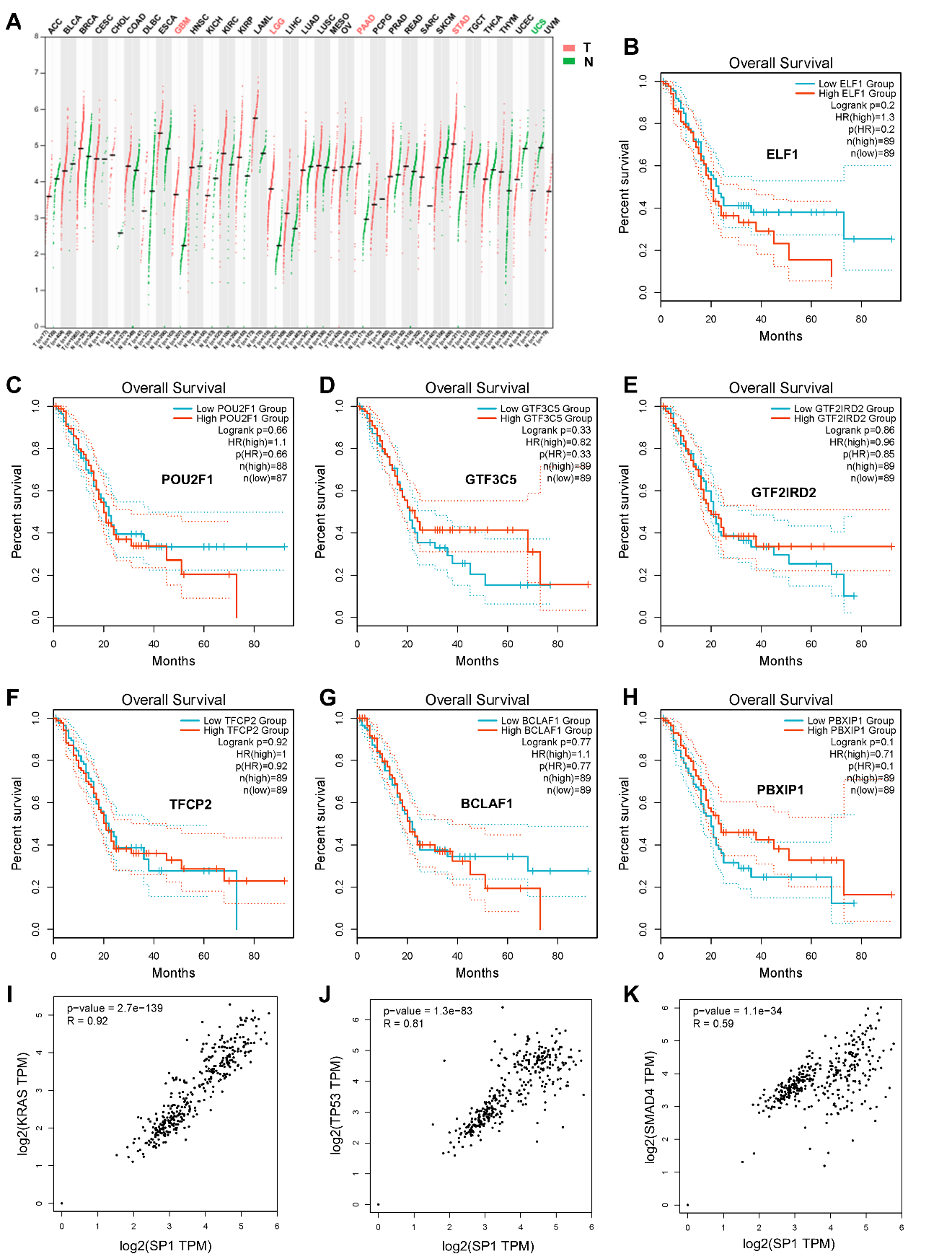


**Supplementary Figure 2.** **SP1 Upregulation in PanINs and PDACs Correlates with Poor Patient Prognosis.**

(A) Expression of SP1 in pancreatic cancer, glioblastoma, stomach adenocarcinoma and other malignant tumors from TCGA and GTEx datasets on GEPIA. (B-H) The other top up-regulated transcriptional factors except SP1 showed no significant correlation with PDAC prognosis. Kaplan-Meier curves showing the relationship between the levels of ELF1, POU2F1, GTF3C5, GTF21RD2, TFCP2, BCLAF1, and PBXIP1 and the overall survival of PDAC patients from TCGA. *P*-value by log-rank test. (I-K) Correlation analysis between the expression levels of SP1 and KRAS, TP53, and SMAD4 from TCGA and GTEx datasets. *P*-value by Spearman test.


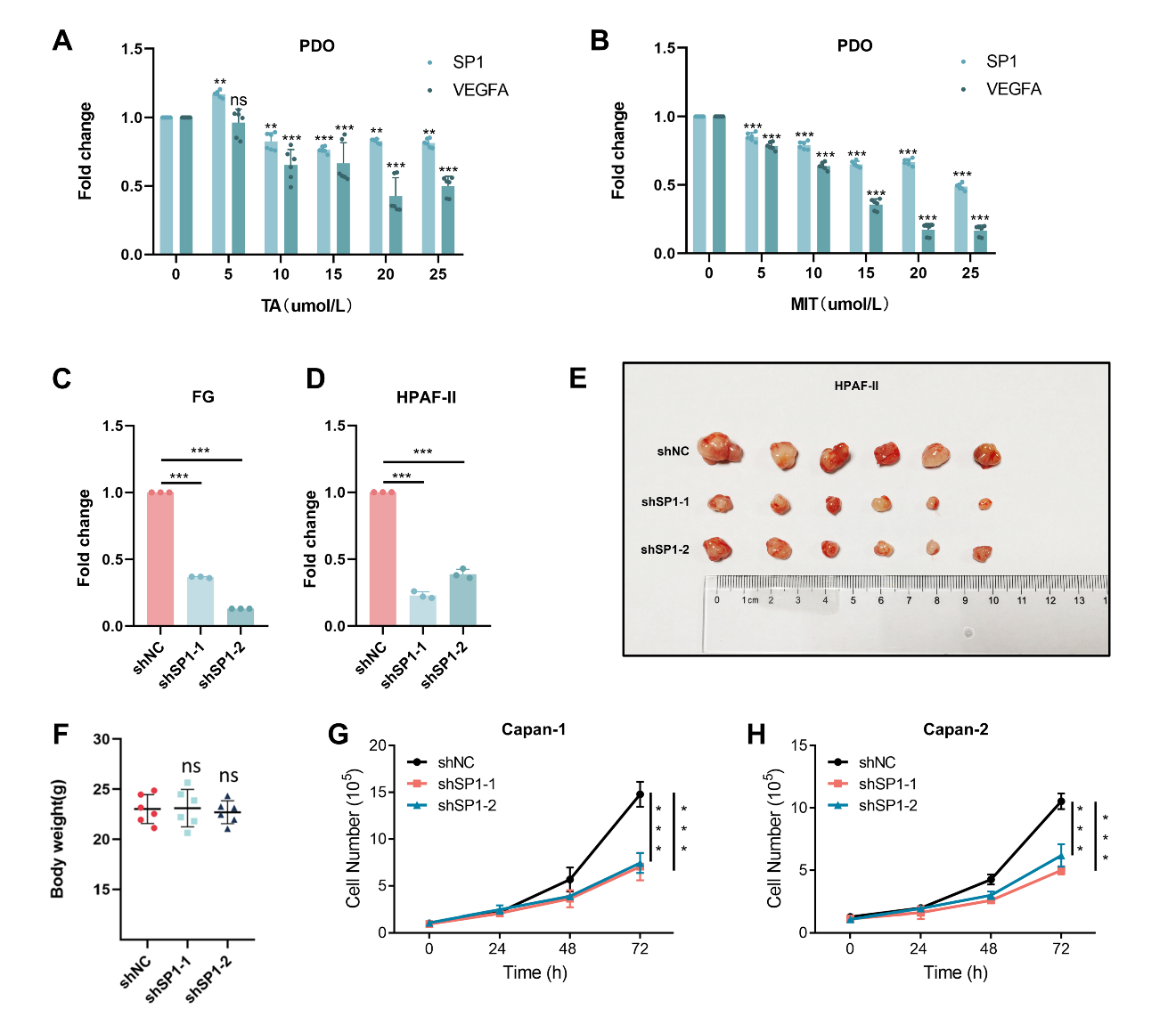


**Supplementary Figure 3.** **SP1 promotes the growth of PDAC cells.**

(A, B) Quantification of the immunoblotting results of SP1 and VEGFA in PDOs treated with MIT or TA using ImageJ. *P*-value by two-way ANOVA test. (C, D) Quantification of the immunoblotting results of SP1 protein levels in control and SP1 knockdown FG and HPAF-Ⅱ cells, as analyzed by ImageJ. *P*-value by one-way ANOVA test. (E) Effect of SP1 knockdown on the growth of HPAF-Ⅱ CDX tumors in vivo (n=6 mice per group). Control and SP1 knockdown cells were orthotopically transplanted onto nude mice, tumors were collected 5 weeks after transplantation. (F) Body weight of nude mice 5 weeks after orthotopically transplantation with FG cells. *P*-value by one-way ANOVA test. (G, H) Growth curve of Capan-1 and Capan-2 cells with or without SP1 knockdown. *P*-value by two-way ANOVA test. Data are shown as mean ± SD. ** *P*＜0.01 and *** *P*＜0.001.


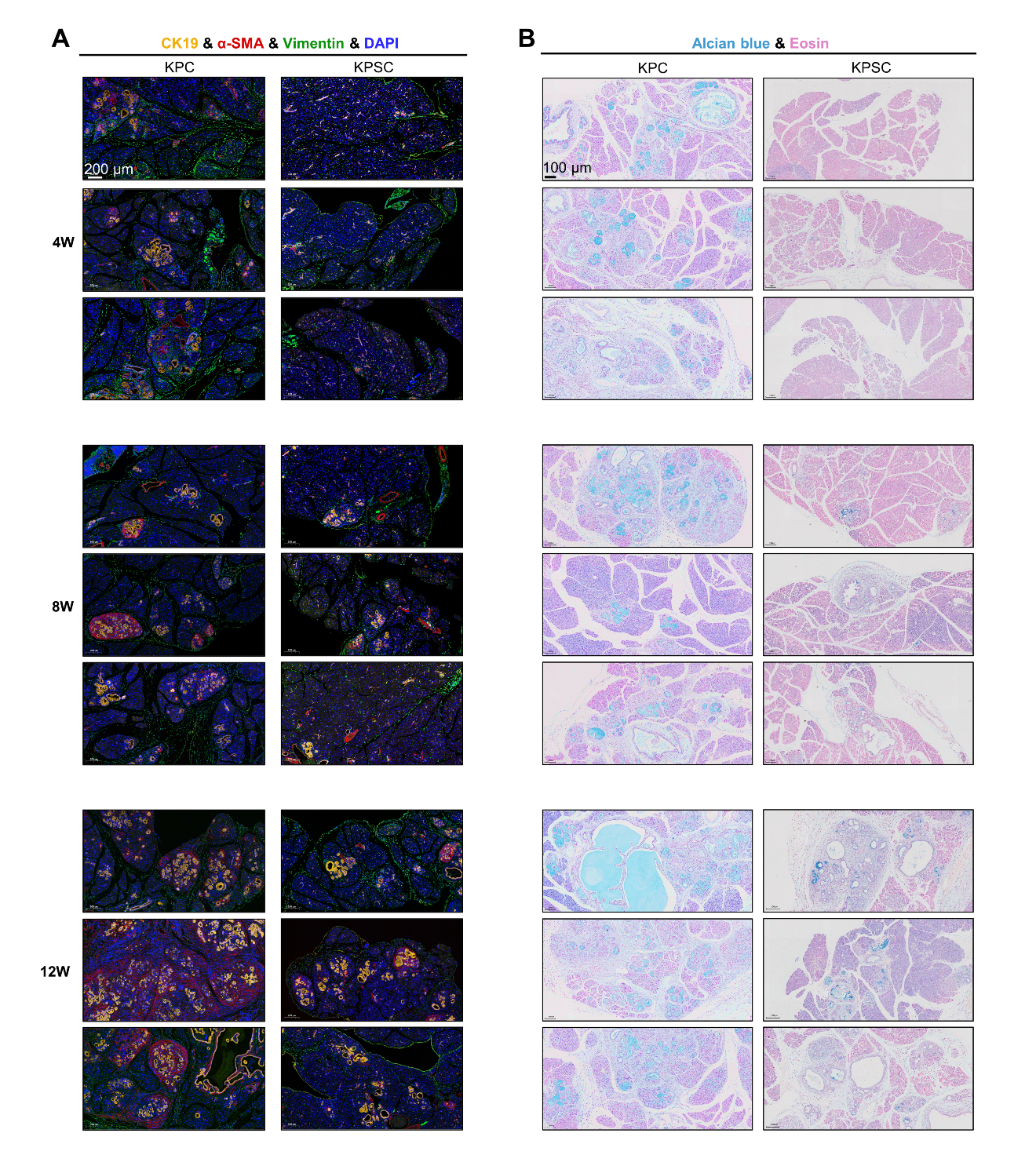


**Supplementary Figure 4.** **Deficiency of Sp1 inhibits tumor development in *Kras^LSL-G12D/+^; Trp53^LSL-R172H/+^; Pdx1-Cre* (KPC) mice.**

(A) Immunofluorescence staining against CK19, α-SMA, and Vimentin in pancreas tissues from KPC and KPSC mice (n=3 mice per group) at 4, 8, and 12 weeks. Scale bars, 200 μm. (B) Alcian blue staining of KPC and KPSC pancreas (n=3 mice per group) at 4, 8, and 12 weeks. Scale bars, 100 μm


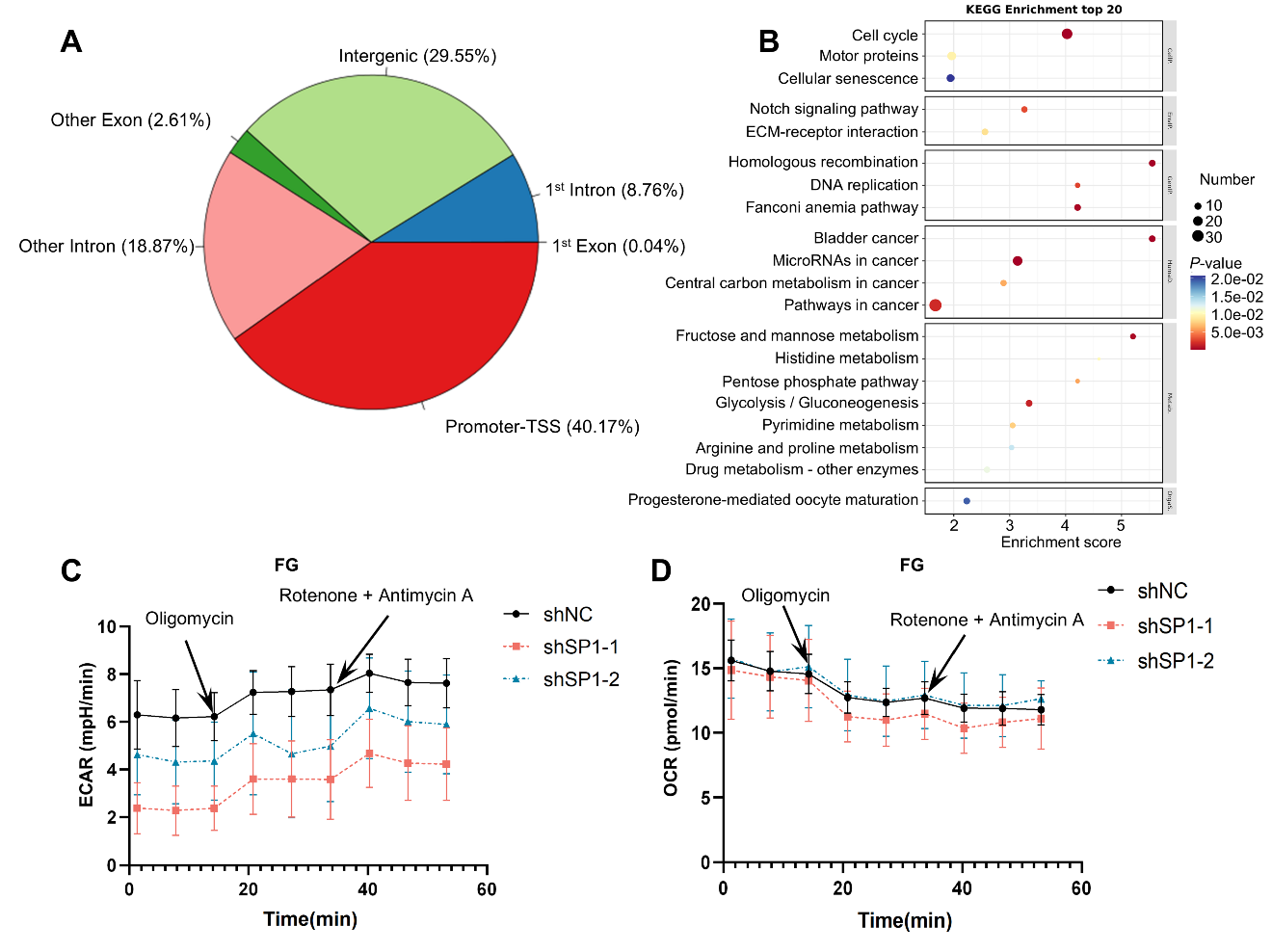


**Supplementary Figure 5.** **SP1 regulates glycolytic metabolism in PDAC cells.**

(A) ChIP-seq experiment for SP1 in FG cells. The distribution of peaks in different gene regions is shown. (B) KEGG enrichment analysis of differentially expressed genes in both ChIP-seq and RNA-seq. (C, D) Seahorse assay results on ECAR (C) and OCR (D) in control and SP1 knockdown FG cells. Data are shown as mean ± SD.


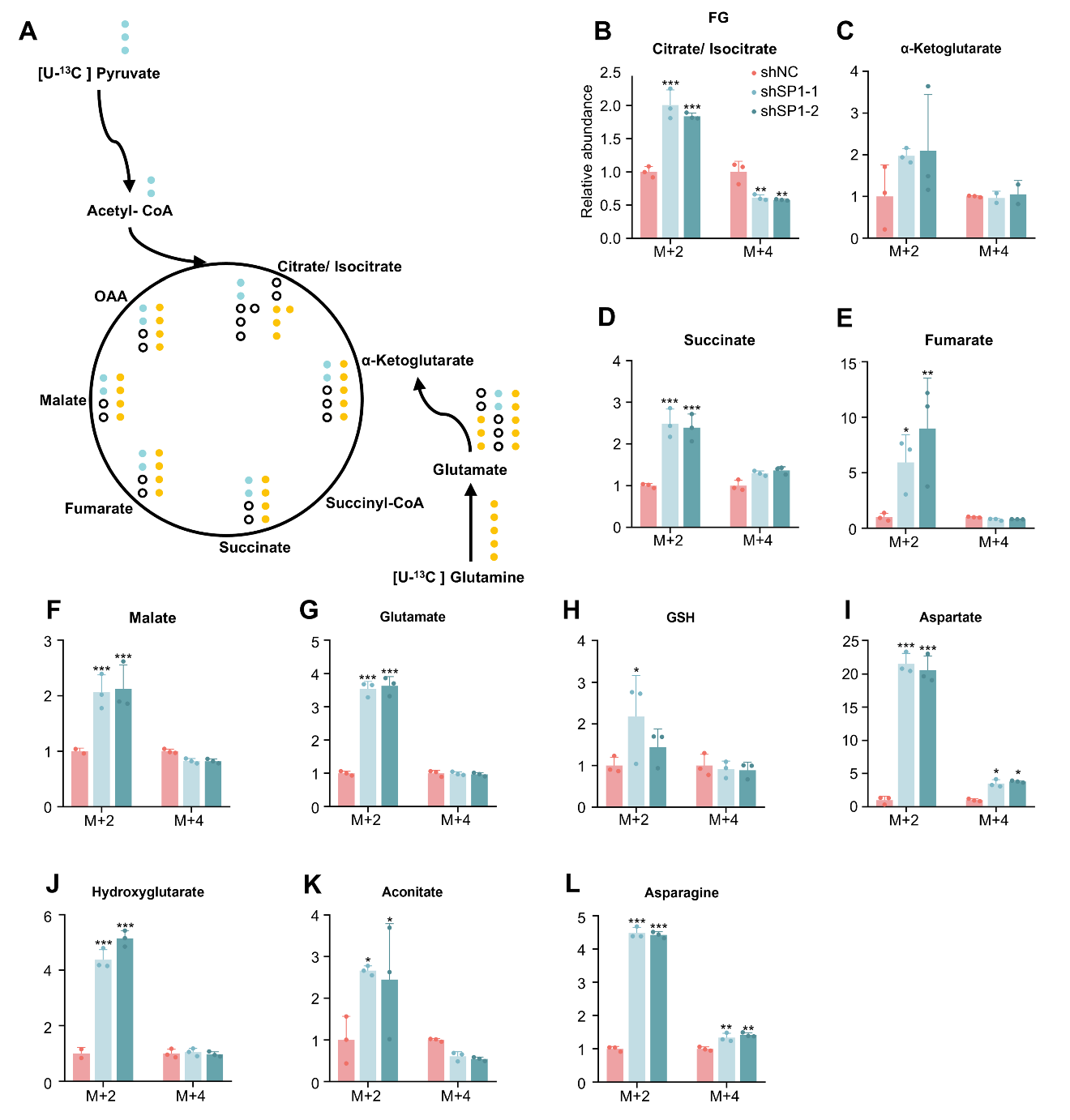


**Supplementary Figure 6.** **Silence of SP1 promotes the utilization of pyruvate in PDAC cells.**

(A) Flow chart of isotope tracing of metabolites in the TCA cycle. (B-L) Quantification of the levels of intermediary metabolites in the TCA cycle by isotope tracing assay. *P*-value by two-way ANOVA test. Data are shown as mean ± SD. * *P*＜0.05, ** *P*＜0.01, *** *P*＜0.001.


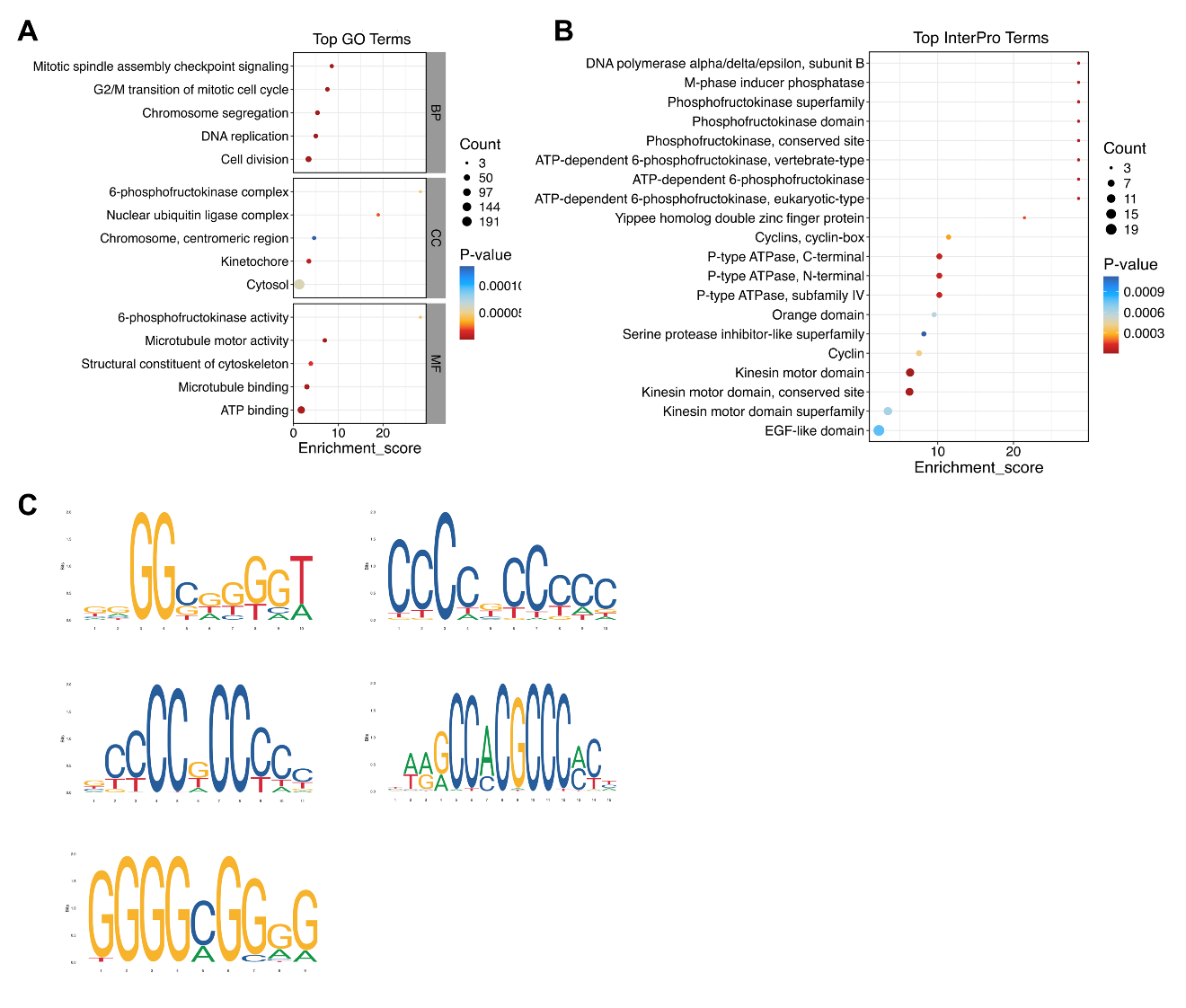


**Supplementary Figure 7.** **SP1 regulates phosphofructokinase in PDAC cells.**

(A, B) GO and InterPro enrichment analysis of differentially expressed genes in both ChIP-seq and RNA-seq. Pathways in regard to phosphofructokinase were significantly enriched in both GO and InterPro analyses. (C) Sequence motifs of the conservative transcriptional binding sites of SP1 from JASPAR database.


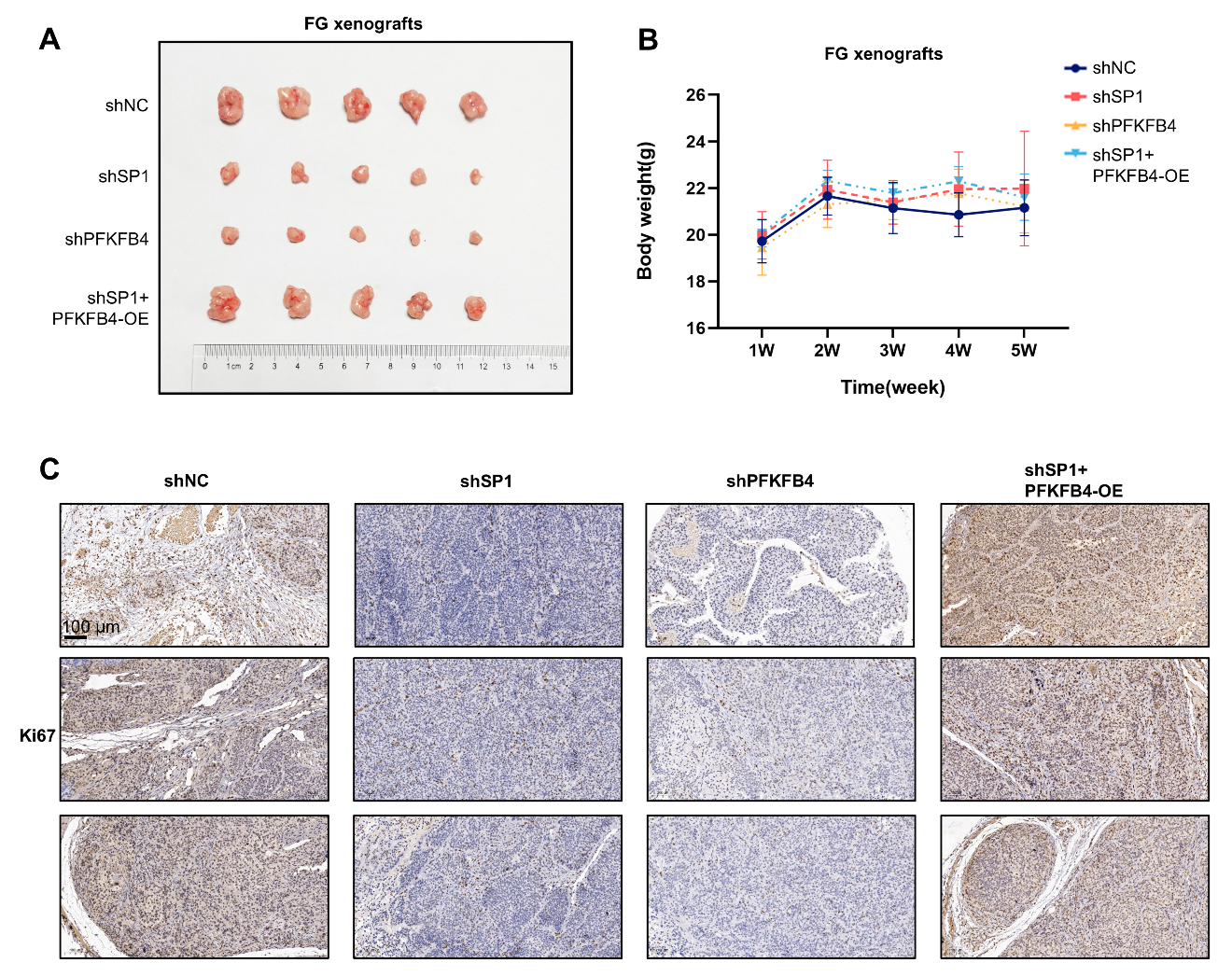


**Supplementary Figure 8. SP1 promotes aerobic glycolysis and growth of PDAC cells through regulating PFKFB4.**

(A-C) PFKFB4 overexpression rescued the suppression of FG CDX tumor growth induced by SP1 or PFKFB4 knockdown. (A) Image of FG CDX tumors orthotopically transplanted onto the pancreas of nude mice. (B) Body weight of nude mice bearing FG CDX tumors. *P*-value by two-way ANOVA test. (C) Ki67 staining of FG CDX tumors. Scale bars, 100 μm. Data are shown as mean ± SD.

**Table S1. Primers used for real-time q-PCR**

| Target  gene | Primer sequence (5’-3’) | |
| --- | --- | --- |
|  | Forward | Reverse |
| ACTB | CATGTACGTTGCTATCCAGGC | CTCCTTAATGTCACGCACGAT |
| SP1 | GTGGAGGCAACATCATTGCTG | GCCACTGGTACATTGGTCACAT |
| PFKM | AGCTGCCTACAACCTGGTGA | TCCACTCAGAACGGAAGGTGT |
| PFKL | GGCTTCGACACCCGTGTAA | CGTCAAACCTCTTGTCATCCA |
| PFKP | CGCCTACCTCAACGTGGTG | ACCTCCAGAACGAAGGTCCTC |
| PFKFB1 | GGCCAGTATCGACGAGAGG | CAAAAACCGCAACATGACCTTC |
| PFKFB2 | ATGACCAACTCCCCGACTCT | TGGACACGTAGGTTTTACCCC |
| PFKFB3 | ATTGCGGTTTTCGATGCCAC | GCCACAACTGTAGGGTCGT |
| PFKFB4 | CAACATCGTGCAAGTGAAACTG | GACTCGTAGGAGTTCTCATAGCA |
| HK1 | CACATGGAGTCCGAGGTTTATG | CGTGAATCCCACAGGTAACTTC |
| HK2 | GAGCCACCACTCACCCTACT | ACCCAAAGCACACGGAAGTT |
| POGLUT1 | AGGACAAGTCCAGAACGAGAT | AGCTACGCCTCGAAAATTAAACA |
| PDHA1 | TGGTAGCATCCCGTAATTTTGC | ATTCGGCGTACAGTCTGCATC |
| PDHB | GCAGCAGTGCTATCTAAAGAAGG | CCAGGAAATTGAACGCAGGAC |
| PDHX | TTGGGAGGTTCCGACCTGT | CAACCACTCGACTGTCACTTG |
| LDHA | CTCCAAGCTGGTCATTATCACG | AGTTCGGGCTGTATTTTACAACA |
| LDHB | CCTCAGATCGTCAAGTACAGTCC | ATCACGCGGTGTTTGGGTAAT |
| GAPDH | GGAGCGAGATCCCTCCAAAAT | GGCTGTTGTCATACTTCTCATGG |
| PGAM1 | GTGCAGAAGAGAGCGATCCG | CGGTTAGACCCCCATAGTGC |
| PGK1 | TTAAAGGGAAGCGGGTCGTTA | TCCATTGTCCAAGCAGAATTTGA |
| GPI | TTTTCCTGTGATGGTGCTTTATGT | AGTTGCCGTCCTGACTGATTGTG |
| ENO1 | CTGGTGCCGTTGAGAAGGG | GGTTGTGGTAAACCTCTGCTC |
| PKM | TGCAGCACCTGATAGCTCG | GGCTTCCATGAGGTCTGTGG |
